# Immobilized enzyme cascade for targeted glycosylation

**DOI:** 10.1038/s41589-023-01539-4

**Published:** 2024-02-06

**Authors:** Elli Makrydaki, Roberto Donini, Anja Krueger, Kate Royle, Ignacio Moya Ramirez, Douglas A. Kuntz, David R. Rose, Stuart M. Haslam, Karen M. Polizzi, Cleo Kontoravdi

**Affiliations:** 1 Department of Chemical Engineering, Imperial College London, London, UK; 2 Department of Life Sciences, Imperial College London, London, UK; 3 Departamento de Ingeniería Química, Universidad de Granada, Granada, Spain; 4 Princess Margaret Cancer Centre, University Health Network, Toronto, Ontario Canada; 5 Department of Biology, University of Waterloo, Waterloo, Ontario Canada

**Keywords:** Glycobiology, Chemical modification, Enzymes, Post-translational modifications

## Abstract

Glycosylation is a critical post-translational protein modification that affects folding, half-life and functionality. Glycosylation is a non-templated and heterogeneous process because of the promiscuity of the enzymes involved. We describe a platform for sequential glycosylation reactions for tailored sugar structures (SUGAR-TARGET) that allows bespoke, controlled N-linked glycosylation in vitro enabled by immobilized enzymes produced with a one-step immobilization/purification method. We reconstruct a reaction cascade mimicking a glycosylation pathway where promiscuity naturally exists to humanize a range of proteins derived from different cellular systems, yielding near-homogeneous glycoforms. Immobilized β-1,4-galactosyltransferase is used to enhance the galactosylation profile of three IgGs, yielding 80.2–96.3% terminal galactosylation. Enzyme recycling is demonstrated for a reaction time greater than 80 h. The platform is easy to implement, modular and reusable and can therefore produce homogeneous glycan structures derived from various hosts for functional and clinical evaluation.

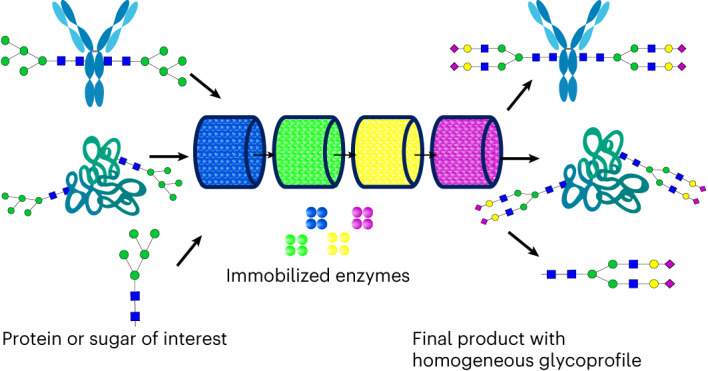

## Abstract (web summary)

SUGAR-TARGET is a modular platform for the homogeneous synthesis of enzymes with controlled N-linked glycosylation using a one-step immobilization/purification method.

## Main

Glycosylation is an essential and complex post-translational modification defining biological and physiological properties of proteins such as folding, stability, activity and trafficking^1^. Therefore, glycosylation is defined as a critical quality attribute of protein-based therapeutics as it directly impacts pharmacological properties of biopharmaceuticals^2^. One example is the N-linked glycans in the Fc domain of monoclonal antibodies, which control receptor binding and affect functionality^3–5^. However, N-linked glycan structures are often heterogeneous, hampering studies of individual structures and limiting our understanding regarding their biological role. Crucially, defining and controlling the desired glycan composition as part of the quality target product profile of protein-based drugs ensures safety and efficacy^6^. The desire to harness the beneficial properties of glycosylation and synthesize proteins with defined glycoforms has led to several efforts in the field of glycoengineering^7–10^.

A common glycoengineering approach involves genetic modification of the native glycosylation pathways of host cells, primarily eukaryotic cells, such as Chinese hamster ovary (CHO), plant and insect cells^10^. Genes of interest are deleted or overexpressed or genes encoding non-native enzymes/pathways are introduced. As a result, proteins with a human-like glycosylation profile have been successfully produced or desired traits such as galactosylation or afucosylation can be enhanced^11,12^. With the evolution of the genetic toolbox, it is also possible to humanize microbial strains such as yeast (for example, *Pichia pastoris*^13^) or introduce de novo glycosylation pathways into bacterial strains such as *Escherichia coli*^14,15^. However, engineering cells for bespoke functions is a time-consuming strategy involving design and complex genome engineering for hosts to produce the desired glycans and identification of the right clone. Modifying native glycosylation pathways can interfere with cellular function, including growth, leading to increased costs and scale-up limitations^16^. Furthermore, the lack of strict control over pathway manipulation and the availability of enzymes and sugar donors still leads to a heterogeneous glycoprofile and can generate off-target glycans or unknown linkages that may be immunogenic or potentially limit biotherapeutic applications^16–18^.

In vitro glycoengineering allows for strict control over reaction conditions and hence glycosylation profiles. Chemoenzymatic methods, with the en bloc transfer of preassembled glycoforms to an appropriately modified protein, produce site-specific and homogeneous sugar structures^19–21^. However, these methods are laborious and produce undesirable chemical byproducts. In vitro enzymatic treatment is a simple alternative, but there are important limitations, including a lack of homogeneity due to enzyme competition, undesired enzyme cross-reactivity and the need to purify the target protein, leading to loss of material and enzyme^22^.

Despite the obvious progress, glycoengineering remains challenging given the complex underlying reaction network, its interaction with cell metabolism and the lack of control over reaction conditions. Furthermore, glycosylation enzymes are known to be promiscuous and recognize and compete for multiple substrates, which results in a high degree of glycan heterogeneity^23,24^. This heterogeneity complicates the large-scale and bespoke application of proteins for use as therapeutics, as the beneficial properties known to be offered by specific homogeneous glycoforms are not always ensured.

**Fig. 1:**
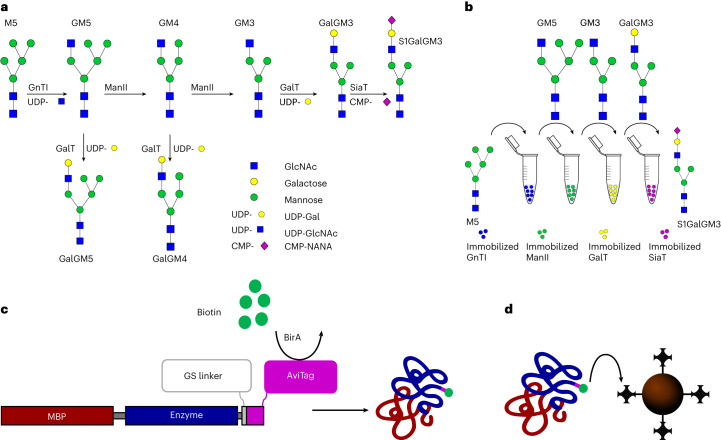
Strategy and design of artificial Golgi reactions (SUGAR-TARGET) for targeted glycosylation. **a**, N-Linked glycosylation pathway as regulated by GnTI, ManII, GalT and SiaT. GalT simultaneously recognizes the products of GnTI and ManII, making it a highly promiscuous enzyme. **b**, Sequential glycosylation reactions using immobilized enzymes. The facile enzyme recovery and thus the spatiotemporal separation will yield the desired product, S1GalGM3, in a homogeneous state. **c**, In vivo biotinylation strategy. The catalytic domain of a target enzyme is fused to an MBP on the N terminus and AviTag on the C terminus. To ensure functionality, a small two-residue glycine–serine (GS) linker was inserted before the AviTag. The biotin ligase BirA recognizes AviTag and can perform enzymatic biotinylation. **d**, Biotinylated enzyme is subsequently immobilized on streptavidin-coated solid supports.

To address the limitations of existing glycoengineering techniques, we developed a platform for artificial Golgi reactions (sequential glycosylation reactions for tailored sugar structures (SUGAR-TARGET)) based on a previously proposed theoretical design^25^. The SUGAR-TARGET platform comprises immobilized enzymes, enabling sequential reactions in an in vitro environment. To achieve immobilization of glycosyltransferases, we developed a strategy encompassing in vivo biotinylation^26,27^ followed by one-step immobilization/purification. The spatiotemporal separation of SUGAR-TARGET addresses existing limitations associated with enzyme promiscuity, allowing bespoke and homogeneous N-linked glycosylation of proteins. Additionally, our platform offers the ability to easily synthesize different target structures, without any chemical modifications, while overcoming existing challenges of in vivo production. To demonstrate the versatility and potential of our platform, we performed several proof-of-concept experiments. We selected a human-like glycosylation pathway encoded by four enzymes, *N*-acetylglucosaminyltransferase I (GnTI)^28^, α-mannosidase II (ManII)^29^, β-1,4-galactosyltransferase (GalT)^30^ and β-galactoside α-2,6-sialyltransferase I (SiaT)^31^, where enzyme competition naturally occurs (Fig. 1a). We successfully tested the enzymatic cascade using the immobilized enzymes on free artificial glycans to produce homogeneous glycoforms (Fig. 1b). We then demonstrated the versatility of our platform by applying the cascade on a monomeric antibody fragment (mFc) expressed in glycoengineered *P. pastoris* and on saposin B produced in a glycoengineered human embryonic kidney (HEK) HEK293 GnTI^–/–^ cell line. In addition, we used immobilized GalT to drive the terminal galactosylation of IgGs from various sources, yielding therapeutically desirable glycoforms. Finally, we demonstrated reusability by recycling the immobilized GalT with a combined reaction time greater than 80 h. These results highlight the potential of SUGAR-TARGET to precisely control the glycosylation profile of a multitude of protein targets derived from microbial and mammalian sources and pave the way for the rapid generation of homogenous glycoprotein structures for functional and clinical evaluation.

## Results

### Immobilization of active glycosyltransferases

**Fig. 2:**
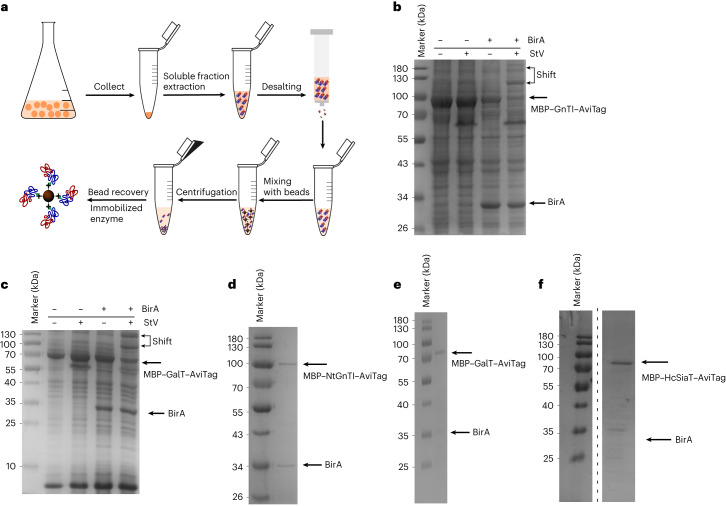
Experimental process for in vivo biotinylation and one-step immobilization/purification of GnTI and GalT. **a**, Following cell collection, cells were lysed via sonication, and the soluble contents, including the enzymes, were separated via centrifugation and desalted. The desalted solution was mixed with streptavidin particles to capture the biotinylated enzyme. A final centrifugation step allowed for the recovery of the immobilized enzyme, while the unbound material was discarded. **b**, Biotinylation confirmation for GnTI using a gel shift assay. Each lane was loaded with and without BirA and streptavidin in the absence of reducing agent (DTT). The molecular weight of MBP–GnTI–AviTag is 93.8 kDa, and the molecular weight of MBP–GalT–AviTAg is 76.4 kDa. **c**, Biotinylation confirmation for GalT using a gel shift assay. Each lane was loaded with and without BirA and streptavidin in the absence of reducing agent. **d**, GnTI recovery after one-step immobilization/purification. BirA binds non-specifically. **e**, GalT recovery after one-step immobilization/purification of GalT. **f**, SiaT recovery after one-step immobilization/purification (the molecular weight is 76.8 kDa); StV, streptavidin. Source data

To develop SUGAR-TARGET, we first expressed three glycosyltransferases (GnTI, GalT and SiaT) in a bacterial host (*E. coli*) and directly immobilized the enzymes from the soluble cell lysate without purification first. These enzymes catalyze an N-linked glycosylation pathway where substrate competition is naturally present (Fig. 1a). The pathway starts with GnTI, followed by ManII and GalT, both of which compete for the same substrate (GlcNAcMan5GlcNAc_2_ (GM5)). For the immobilization method, we chose the biotin–streptavidin complex given its high stability and ability to form under mild conditions. Furthermore, to exploit the high protein yields from expression in *E. coli*, we implemented an enzymatic in vivo biotinylation method based on the BirA/AviTag interaction^26,27^. Specifically, the genes encoding GnTI (Δ29) from *Nicotiana tabacum*, human GalT (Δ128) and *Helicobacter cetorum* 2,6-SiaT (Δ42, C terminus^31^) were fused to maltose-binding protein (MBP) at the N terminus to achieve soluble expression in *E. coli* and AviTag at the C terminus through a small, two-residue glycine–serine linker, allowing site-specific immobilization (Fig. 1c). This design enables the one-step immobilization/purification of glycosyltransferases (Figs. 1d and 2a).

**Extended Data Fig. 1:**
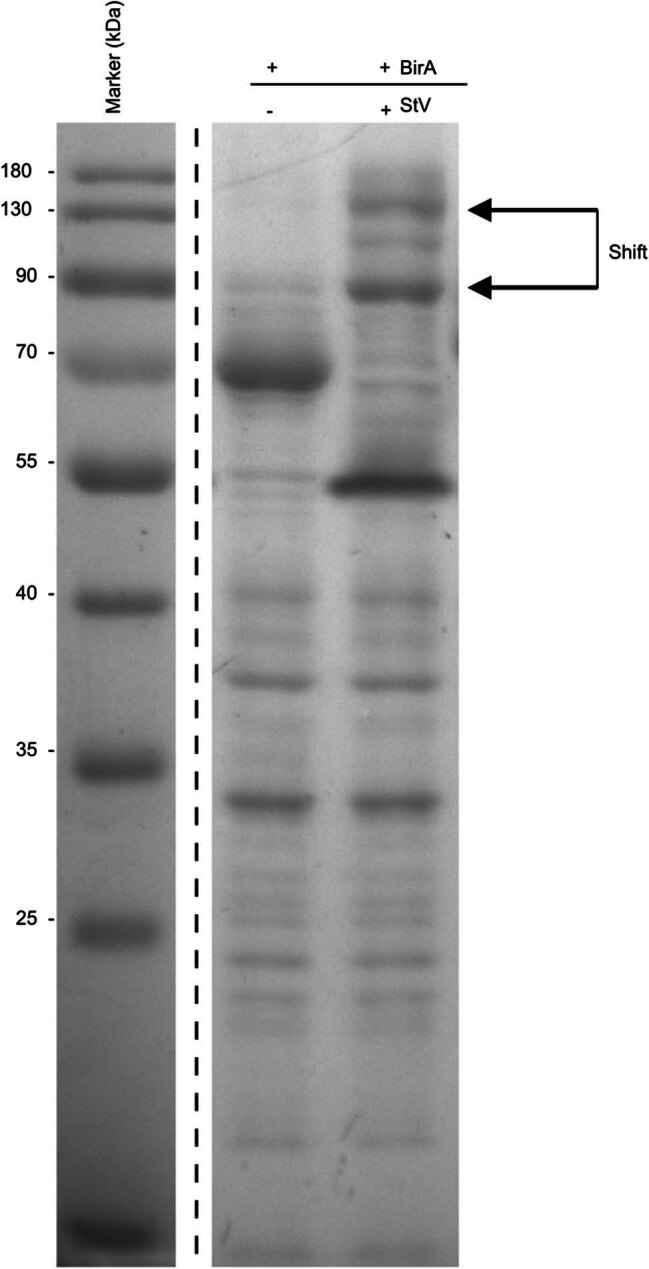
Biotinylation confirmation for SiaT using a gel shift assay. Each lane was loaded with and without StV in the absence of reducing agent (DTT). Source data

Coexpression with BirA and medium supplementation with biotin after induction led to successful site-specific biotinylation of GnTI, GalT and SiaT. For confirmation, a gel shift assay using free streptavidin and the soluble fraction following cell lysis was performed as previously described^27^. Image analysis showed that more than 65% of both GnTI and GalT and more than 85% of SiaT were biotinylated when 20 µM biotin was added to the culture medium (Fig. 2b,c and Extended Data Fig. 1). Interestingly, when performing the gel shift assay, two new distinct bands were detected. Considering that streptavidin has a valency of 4, the multiple bands likely correspond to different streptavidin–enzyme complexes. Following expression, we removed free biotin via a desalting step and subsequently incubated the desalted samples with streptavidin-coated silica beads. One-step immobilization/purification was confirmed using gel electrophoresis for all glycosyltransferases (Figs. 2d–f). BirA also binds non-specifically to the beads, possibly due to the formation of a complex with its substrate AviTag.

**Extended Data Fig. 2:**
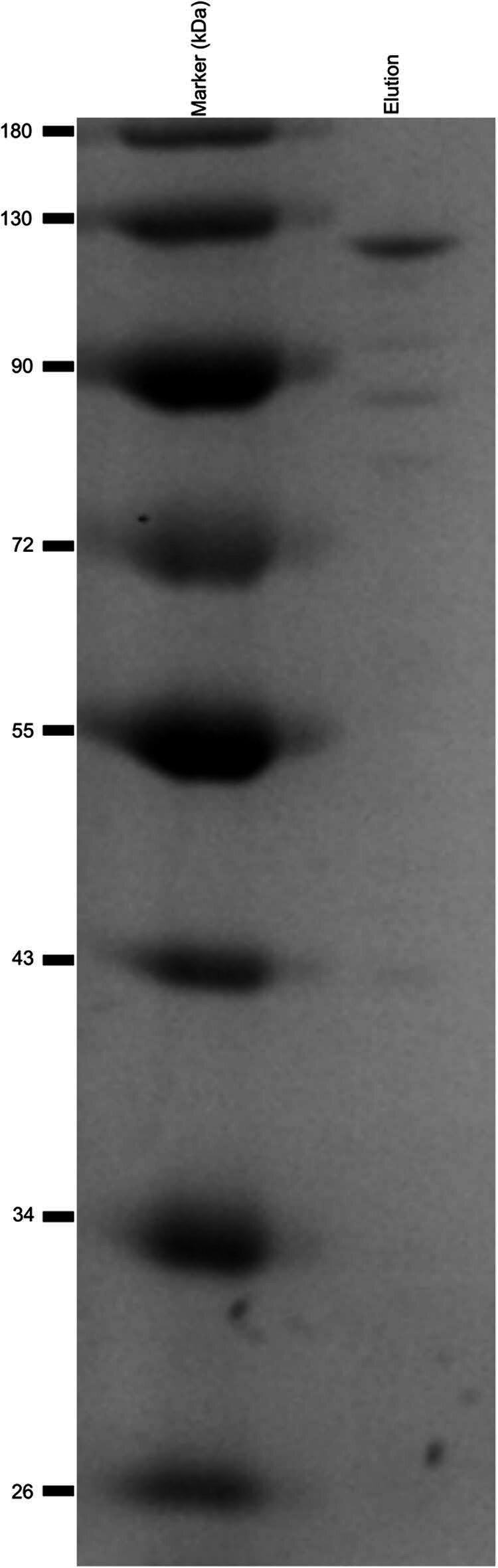
SDS-PAGE analysis of enzyme recovered from the streptavidin particles following immobilisation of chemically biotinylated ManII. Source data

Attempts to express and purify ManII in *E. coli* were not successful. Therefore, purified *Drosophila melanogaster* ManII produced in insect cells was biotinylated in vitro using a chemical biotinylation technique. It was subsequently immobilized on streptavidin-coated beads as confirmed by SDS–PAGE analysis (Extended Data Fig. 2).

**Extended Data Fig. 3:**
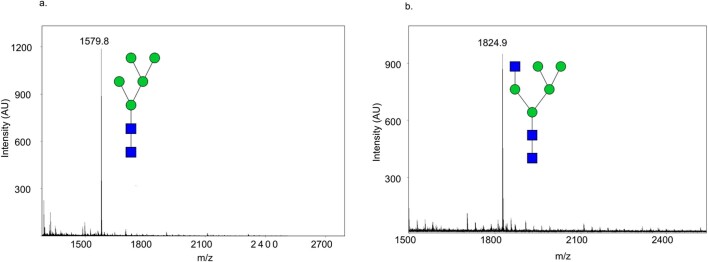
Confirmation of activity of immobilised GnTI as monitored by MALDI-TOF MS. **a**. 0 h, acceptor sugar M5 (m/z = 1579) **b**. overnight GnTI reaction. Reaction product GM5 (m/z = 1824).

**Extended Data Fig. 4:**
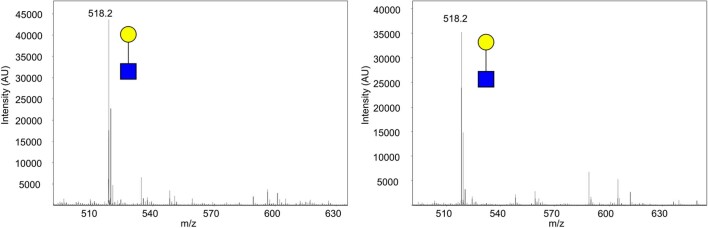
Confirmation of activity of immobilised GalT as monitored by MALDI-TOF MS. The monosaccharide GlcNAc was used as a starting substrate. Due to the difficulty in detecting single sugars such as GlcNAc with MALDI-TOF MS methodology, it was not feasible in this experimental set-up, to quantify the extent of the reaction and determine whether it was near completion.

**Extended Data Fig. 5:**
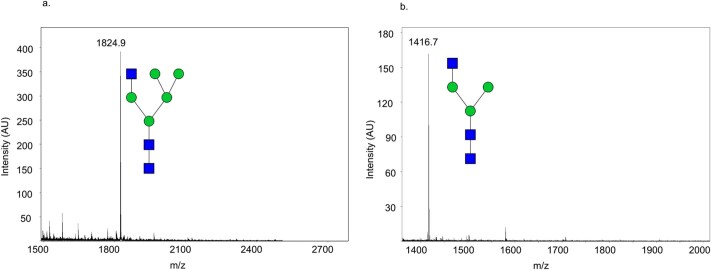
Reactions of immobilised ManII as monitored by MALDI-TOF MS. **a**. 0 h, starting sugar GM5 (m/z 1824); **b**. Overnight ManII reaction. Reaction product GM3 (m/z = 1416).

**Extended Data Fig. 6:**
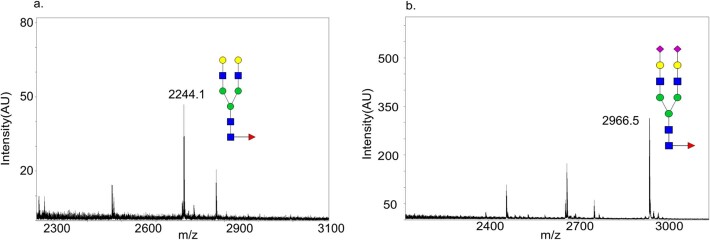
Reactions of immobilised SiaT as monitored by MALDI-TOF MS. **a**. 0 h, starting sugar G2F (m/z 2244); **b**. Overnight SiaT reaction. Reaction product S2G2F (m/z = 2966).

Following successful implementation of the biotinylation and immobilization schemes, the activity of immobilized GnTI, ManII, GalT and SiaT was confirmed by MALDI-TOF MS/MS analysis (Extended Data Figs. 3–6) using model reactions with suitable free and protein-bound glycan substrates under previously described conditions^28–30^. Specifically, GnTI was reacted with M5 (Man5GlcNAc2) to produce GM5, ManII with GM5, GalT with free *N*-acetylglucosamine (GlcNAc) and SiaT with G2F (GlcNAcMan3GlcNAc2Gal2 with core fucose, a biantennary glycan). These results confirmed that biotinylation (in vivo or in vitro) and subsequent immobilization did not hinder the catalytic activity of these enzymes.

### Synthesis of pure free glycoforms

**Extended Data Fig. 7:**
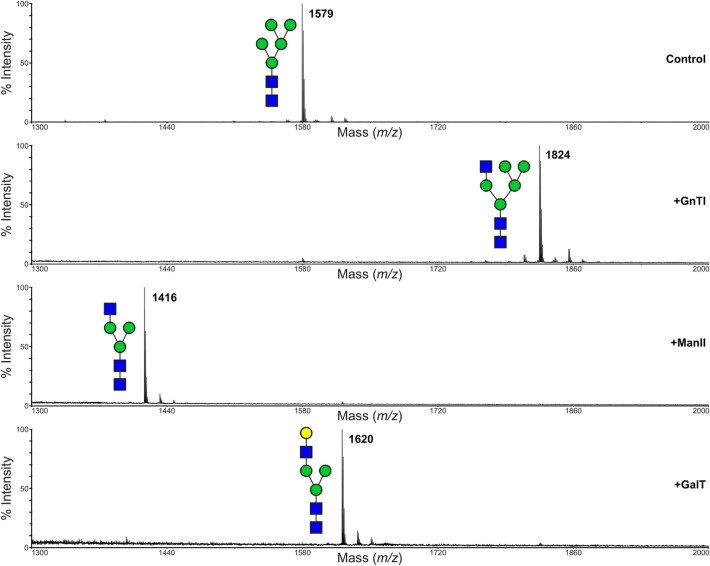
MALDI-TOF MS profile of permethylated N-linked glycans monitoring the immobilised enzyme cascade on free glycans. The first panel shows the starting substrate M5 without enzymatic processing (control). Subsequent panels show the glycomic profile of M5 after sequential processing in SUGAR-TARGET with GnTI, ManII and GalT. All molecular ions represent the singly charged and sodiated form ([M + Na]^+^).

Following successful biotinylation and immobilization of GnTI, ManII and GalT on silica beads, we assembled our SUGAR-TARGET platform and tested its ability to produce homogenous glycans. Our first test case was the production of homogeneous free glycans. These polysaccharides have several applications, such as in the characterization of enzyme–substrate interactions and glycan-binding proteins, such as lectins or antibodies, and in enzyme kinetic studies. However, pure glycans with different structures are difficult to isolate or synthesize on demand^10^. Therefore, we applied the enzymatic cascade aiming for near-pure glycans after each step (Fig. 1a). At the end of each reaction, the immobilized enzyme was removed via centrifugation before the addition of the next enzyme in the cascade. All steps were individually monitored by using MALDI-TOF MS analysis (Extended Data Fig. 7). As such, immobilized GnTI was reacted with the free glycan M5 to produce the glycan GM5 (>95% conversion). GM5 was then reacted with immobilized ManII in its corresponding buffer to successfully remove the α-1,6- and α-1,3-mannose residues. As confirmed by MALDI-TOF MS analysis, conversion of the glycan GM5 to GM3 was also >95%. GM3 was subsequently galactosylated using immobilized GalT. The desired product GalGM3 of the enzymatic pathway GnTI–ManII–GalT was produced, synthesizing a near-pure glycoform (>95% conversion). Our results demonstrate that the specificity of all three enzymes was near 100%, enabling SUGAR-TARGET to be used as an on-demand platform to produce desired glycoforms.

### Humanization of recombinant glycoproteins

Following the successful implementation of SUGAR-TARGET on free glycans, we sought to demonstrate the applicability of our platform to different glycosylated proteins of therapeutic interest derived from various glycoengineered hosts. We applied our GnTI–ManII–GalT–SiaT cascade to an mFc crystallizable domain produced in the glycoengineered *P. pastoris* strain SuperMan5 (GlycoSwitch technology) and on saposin B produced in glycoengineered HEK293 cells lacking GnTI activity (GlycoDelete)^32^. Both types of glycoengineered cells produce predominantly M5 structures^16,33^, the preferred substrate for GnTI. The aim was to demonstrate that SUGAR-TARGET can be used to modify a range of cell-derived material, allowing for a multitude of future applications, such as drug efficacy studies, for example, studying the effect on antibody–receptor binding and the production of bespoke therapeutics or biosimilars.

**Fig. 3:**
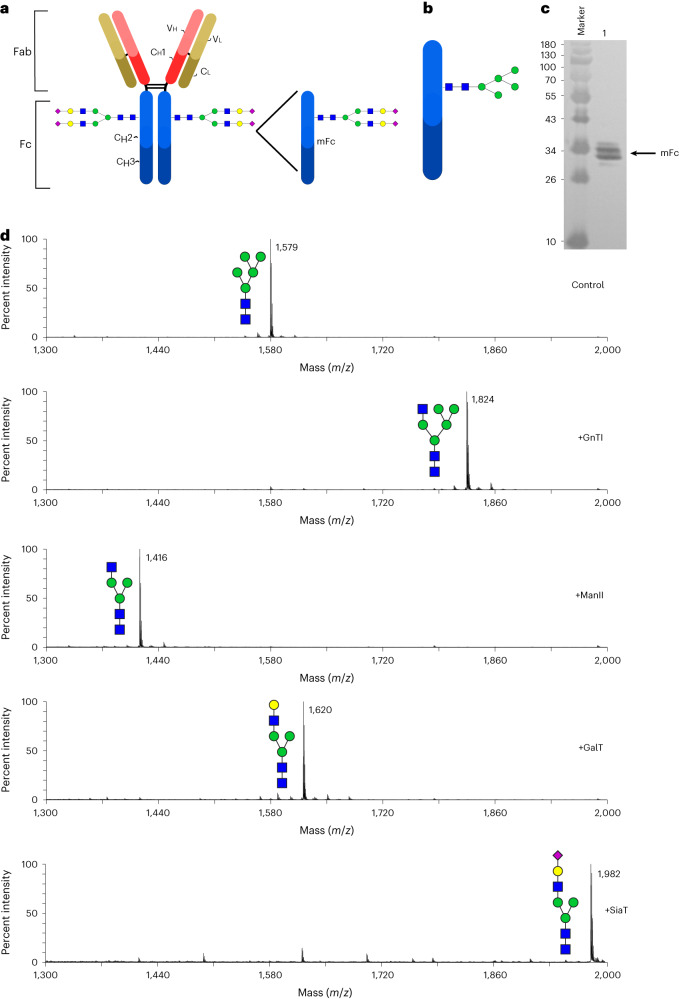
Immobilized enzyme cascade on an mFc. **a**,**b**, Structures of full-length IgG monoclonal antibody (**a**) and mFc (**b**). **c**, mFc produced in the *P. pastoris* strain SuperMan5 to produce mainly M5 structures. **d**, MALDI-TOF MS profile of permethylated N-linked glycans derived from SuperMan5 *P. pastoris*-produced mFc. Top, the glycomic spectrum of the mFc without enzymatic processing (control). Middle and bottom, glycomic profiles of the mFc N-glycans after sequential processing in SUGAR-TARGET with GnTI, ManII, GalT and SiaT. All molecular ions represent the singly charged and sodiated form ([M + Na]^+^); Vh, variable heavy chain; Vl, variable light chain; Ch1, constant heavy chain 1; Cl, constant light chain; Ch2, constant heavy chain 2; Ch3, constant heavy chain 3; Fab, antigen binding fragment; Fc, crystallizable fragment. Source data

Antibodies are well known for their biotherapeutic value^34,35^. In this first application, we produced an mFc, a therapeutically relevant protein^36^ with a single glycosylation site, to facilitate the analysis of the glycoforms in *P. pastoris* SuperMan5 to produce M5 structures (Fig. 3a,b). We expressed the mFc with a C-terminal His tag, it was secreted in the supernatant (Fig. 3c), and we immediately purified it using nickel-nitrilotriacetic acid (Ni-NTA; >97% purity as estimated by image analysis). We then applied a four-enzyme cascade, GnTI, ManII, GalT and SiaT, to produce human-like glycoforms (Fig. 3d). We performed some minor modifications of the reaction conditions used in the previous section to prevent protein precipitation and to improve reaction conversion (see Methods for details). In addition, we changed the solid support from silica to magnetic particles to minimize processing times and improve sampling. After each step, SDS–PAGE and image analyses were used to estimate mFc concentration. Finally, each step was monitored and analyzed by MALDI-TOF MS. Our results show that each enzymatic step approached homogeneity with >95% conversion to the desired product (Fig. 3d), confirming that the spatiotemporal separation addresses enzyme competition and minimizes glycan heterogeneity. Our findings therefore confirm that our platform can be used to reconstruct a mammalian N-linked glycosylation pathway on microbial cell-derived therapeutic products.

**Extended Data Fig. 8:**
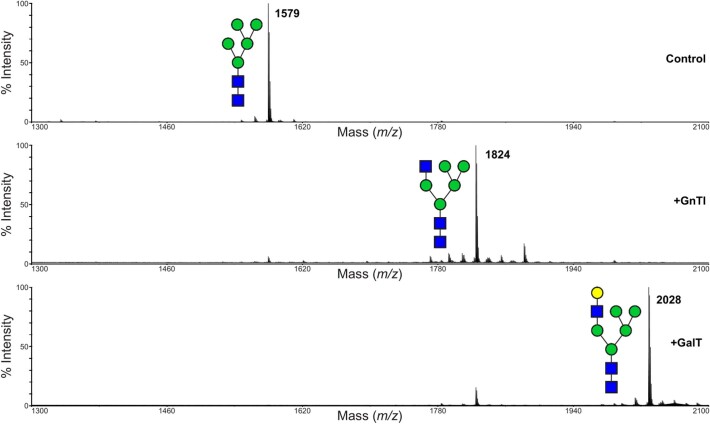
Immobilised GnTI-GalT cascade on monomeric Fc (mFc). MALDI-TOF MS profile of permethylated N-linked glycans derived from SuperMan5 P. pastoris-produced mFc. The first panel shows the glycomic spectrum of the mFc without enzymatic processing (control). Subsequent panels show the glycomic profile of the mFc derived N-glycans after sequential processing in SUGAR-TARGET with GnTI and GalT. All molecular ions represent the singly charged and sodiated form ([M + Na]+); VH: variable heavy chain; VL: variable light chain, CH1: constant heavy chain 1; CL: constant light chain; CH2: constant heavy chain 2; CH3: constant heavy chain 3; Fab: antigen binding fragment; Fc: crystallizable fragment.

To show that we could reconstruct a different mammalian N-linked glycosylation pathway using the same process, we altered the enzymatic pathway to GnTI–GalT aiming to produce GalGM5 as the predominant glycoform (Extended Data Fig. 8). Maintaining the same reaction conditions as before, we achieved over 85% conversion, synthesizing a near-homogeneous GalGM5 glycan intermediate. We anticipate that it will be possible to achieve near 100% conversion with further optimization of the reaction conditions.

Saposins are small non-enzymatic proteins that facilitate the lysosomal degradation of sphingolipids^37^. Although inactive in the absence of lysosomal hydrolases, the interactions between saposins and hydrolases are necessary for their activity and role in immunology, lysosomal disease progression and antimicrobial response^37,38^. The specific role of glycosylation in these interactions is still being explored. The ability to generate homogenous glycoforms can therefore facilitate such functional studies.

**Extended Data Fig. 9:**
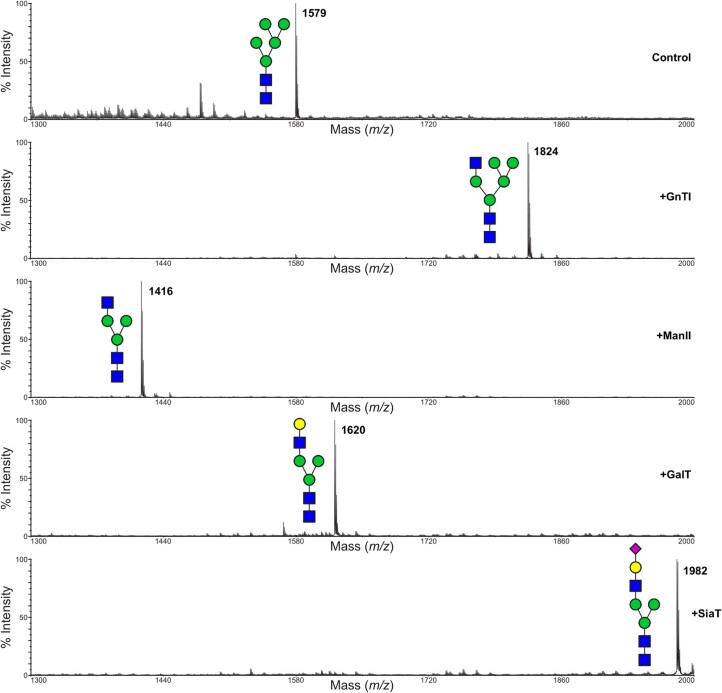
Immobilised enzyme cascade on saposin B. MALDI-TOF MS profile of permethylated N-linked glycans derived from saposin B produced in HEK293 GNTI-/-. The first panel shows the starting substrate M5 without enzymatic processing (control). Subsequent panels show the glycomic profile of M5 after sequential processing in SUGAR-TARGET with GnTI, ManII, GalT and SiaT. All molecular ions represent the singly charged and sodiated form ([M + Na]^+^).

Here, we chose saposin B as a test case to demonstrate the versatility of our platform for producing pure glycan intermediates and applied our four-enzyme cascade to saposin B, which has one glycosylation site. As with our previous results, applying SUGAR-TARGET on saposin B led to highly homogeneous intermediates and final product (>95% conversion to the desired product S1GalGM3; Extended Data Fig. 9), confirming that spatiotemporal separation addresses enzyme promiscuity and glycan heterogeneity.

### Enhancing galactosylation of IgGs using immobilized GalT

**Fig. 4:**
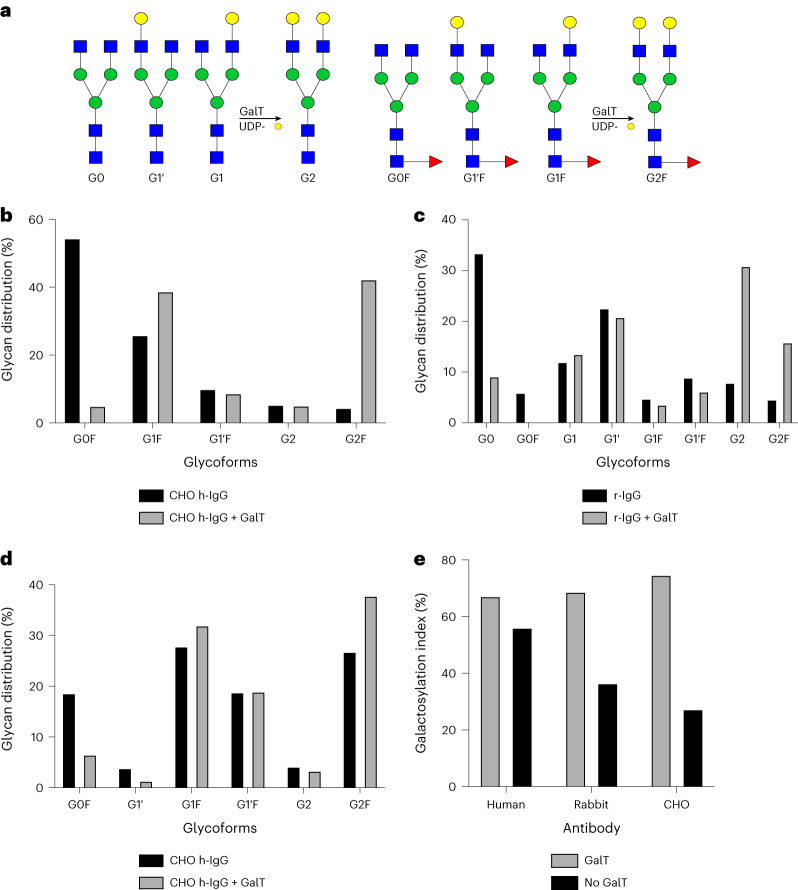
IgG treatment with immobilized GalT. **a**, Biantennary structures formed during treatment with GalT. **b**, Glycoform distribution in CHO h-IgG before and after treatment with immobilized GalT. **c**, Glycoform distribution in r-IgG before and after treatment with immobilized GalT. **d**, Glycoform distribution in h-IgG before and after treatment with immobilized GalT. **e**, Analysis of IgG treatment with GalT (*n* = 2 biological replicates). Glycoforms are compared using the galactosylation index, a measurement of galactose molecules normalized for the maximum number of galactose molecules. Source data

Another application of our technology is the use of immobilized enzymes for altering the galactosylation profile of full-length antibodies, a desirable post-translational modification to improve antibody functionality. Galactose is known to confer anti-inflammatory properties and can enhance receptor affinity, for example, with Fcγ receptors^39–41^. We therefore tested the ability of our in-house immobilized GalT to increase the galactosylation of three IgGs from diverse sources. IgG antibodies from three different sources, humanized monoclonal IgG produced in CHO cells (CHO h-IgG), IgG from rabbit serum (r-IgG) and IgG from human serum (h-IgG), were treated in vitro with immobilized GalT. Terminal galactosylation was analyzed using capillary electrophoresis (CE), and the resulting electropherograms were used to determine the glycoform distribution for each IgG (Fig. 4a). All three IgGs showed increased terminal galactosylation after treatment with the immobilized GalT, and structures galactosylated on both antennae were synthesized in detectable amounts. Remarkably, incubation with immobilized GalT increased terminal galactosylation on CHO cell-derived h-IgG by 93%, resulting in an overall galactose content of 96.3% (Fig. 4b). h-IgG treated with immobilized GalT had a 15% increase in terminal galactosylation from ~70% to ~84% (Fig. 4c). Finally, the reaction of r-IgG with immobilized GalT led to an increase in terminal galactosylation from ~60% to ~91% (Fig. 4d). Our data demonstrate that immobilized GalT can consistently operate on a variety of IgGs, increasing galactosylation to >80% in all cases tested (Fig. 4e).

### Understanding substrate specificity of immobilized GalT

**Fig. 5:**
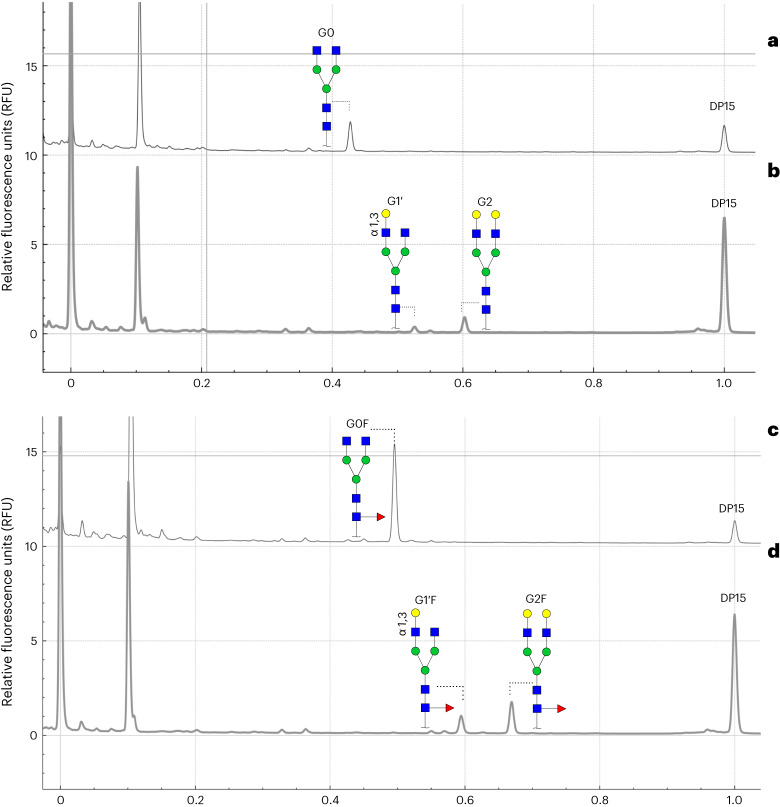
Reaction of immobilized GalT with glycans. **a**, Electropherogram of the starting substrate G0. **b**, Electropherogram of the reaction products of G0 following a 15-min treatment with immobilized GalT. **c**, Electropherogram of the starting substrate G0F. **d**, Electropherogram of the reaction products of G0F following a 15-min treatment with immobilized GalT. Source data

**Extended Data Fig. 10:**
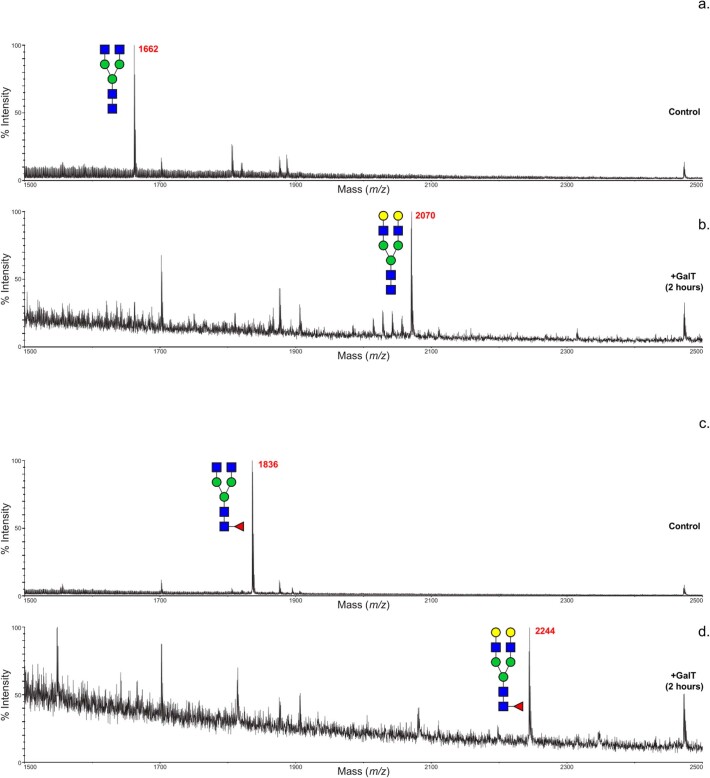
MALDI-TOF MS profile of permethylated N-linked glycans monitoring the activity of GalT on two free N-glycan standards GlcNAc2Man3GlcNAc2 (G0) and GlcNAc2Man3GlcNAc2Fuc (G0F). **a**. untreated G0; **b**. Reaction product of G0 following 2 hour treatment with immobilised GalT. Only the fully galactosylated product G2 is detected.; **c**. untreated G0F; b. Reaction product of G0F following 2 hour treatment with immobilised GalT. Only the fully galactosylated product G2F is detected. Dominant signals at m/z 2682 were identified as contaminants All molecular ions represent the singly charged and sodiated form ([M + Na]+).

Following our analysis of IgG molecules, we wanted to understand the specificity of immobilized GalT toward free glycans. We incubated the enzyme with G0 and G0F (GlcNAcMan3GlcNAc2 and GlcNAcMan3GlcNAc2Fuc, biantennary glycans lacking galactose with and without core fucose, respectively) for 2 h and analyzed the reaction by MALDI-TOF MS. As seen in Extended Data Fig. 10, only G2 and G2F were detected in resulting samples, indicating that the reaction had reached completion. We hypothesized that given the differences in substrate concentration and steric hindrance, the reaction would operate at a faster rate than that observed with the IgG substrate. We therefore incubated our immobilized GalT with G0 and G0F for 15 min to capture intermediate products before full conversion and analyzed the reaction by using CE. Compared to MALDI-TOF MS, CE has the advantage of detecting arm specificity, allowing us to distinguish between G1 and G1′ and between G1F and G1′F. We observed that G0 was mostly converted (>75%) to G2, with some G1′ remaining (Fig. 5a,b). Using G0F instead of G0 yielded similar results (>65% conversion to G2F with G1′F remaining (Fig. 5c,d). These results suggest that the addition of a single galactose on the α-1,3 arm is the limiting step, as previously shown^42^. Finally, we investigated the specificity of GalT toward monogalactosylated free glycans by incubating for 15 min with G1/G1′ and G1F/G1′F (Supplementary Fig. 1). Our results show that the in-house GalT can rapidly modify fucosylated and non-fucosylated glycoforms. In addition, when reacting with fucosylated structures, we observed that GalT modified G1F faster (85% conversion compared to 56% conversion of G1′F) by adding an additional galactose on the α-1,3 arm. These results agree with the sequential addition of galactose initially in the α-1,6 arm, followed by addition in the α-1,3 arm, as seen in our IgG results.

### Enzyme immobilization enables reusability

**Fig. 6:**
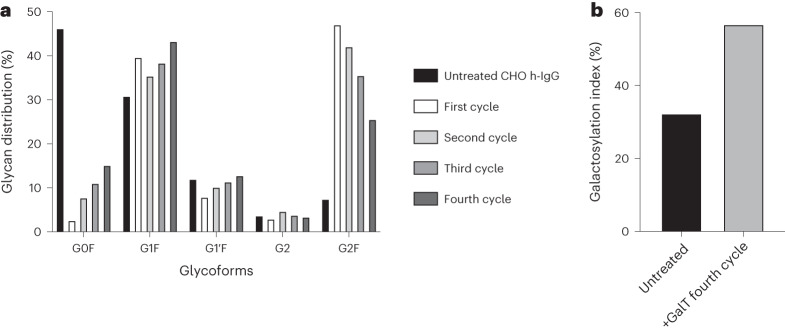
Reusability of immobilized GalT. **a**, Enzyme reusability and glycoform distribution of CHO h-IgG in multiple cycles. Changes in galactosylation depict a linear decrease with each cycle (Pearson correlation coefficient for G0F = 0.94; The results shown are from independent experiments, *n* = 2 biological replicates). **b**, Analysis of untreated IgG and IgG treated after the fourth reusability cycle. Glycoforms were compared using the galactosylation index, a measurement of galactose molecules normalized for the maximum number of galactose molecules (here = 2). Source data

We next evaluated the longevity and reusability of our in-house immobilized GalT to demonstrate the sustainability and industrial deployment potential of the proposed SUGAR-TARGET platform^43^. We focused on GalT because galactosylation is a highly desirable critical quality attribute of glycoprotein therapeutics and therefore is of particular industrial and clinical interest. As our immobilized GalT yielded the largest improvement on the CHO h-IgG substrate, we proceeded with this substrate to conduct enzyme reusability experiments, maintaining the same reaction conditions. Specifically, GalT was used for four consecutive reaction cycles, with recovery and wash steps between each cycle, and results were monitored by CE. Reusability and enhanced galactosylation of CHO h-IgG was successfully demonstrated for all four cycles (Fig. 6). The results show that the enzyme retained over 70% of its activity after 80 h of usage. The small observed decrease in activity can be attributed to the loss of small amounts of enzyme during washes after each cycle. Remarkably, terminally galactosylated structures increased from 52% to 97.4% after the first cycle. After the fourth and final cycle, terminal galactosylation was at 84%. Our results confirm that the use of our immobilized GalT over multiple cycles can produce human-like glycoforms. Therefore, our strategy is an attractive alternative to using free enzymes and can vastly reduce costs associated with downstream processes as well as costs associated with enzyme production^19,43,44^.

## Discussion

It is well-established that glycosylation can modulate biopharmaceutical protein functionality, such as efficacy, half-life and immunogenicity. Therefore, controlling glycosylation is highly desirable to further understand the biological role of glycans and to generate tailored biotherapeutics with bespoke glycosylation. However, glycosylation within cells is highly complex due to the promiscuity of enzymes and the absence of a reaction template, resulting in heterogeneous structures. In this work, we developed SUGAR-TARGET, a platform comprising immobilized enzymes for targeted and sequential glycosylation reactions in an in vitro environment. We demonstrate that our compartmentalized design, a result of enzyme immobilization, can address enzyme promiscuity in a pathway where competition naturally exists, offering strict control over the final glycoprofile.

We initially developed a method for the in vivo biotinylation and site-specific immobilization of glycosyltransferases. We applied this methodology to three glycosyltransferases, GnTI, GalT and SiaT, and achieved over 65% biotinylation. This yield can be further improved by process optimization, for example, by increasing the amount of d-biotin in the culture medium^26,27,45^. Furthermore, we showed that our methodology is independent of the origin of the glycosyltransferase, as we applied it to a variety of enzymes derived from plant, human and bacterial species. One advantage of using enzymatic biotinylation is the simplicity and versatility of the method as it can be applied either in vivo, with coexpression of BirA, or in vitro, where all components are previously purified. This approach does not require excessive enzyme engineering that might negatively affect structure or enzyme activity. Crucially, the methodology is compatible with multiple enzyme expression platforms, such as bacteria, insect, plant or mammalian cell-based systems^27,45–47^. Therefore, in the future, we expect this strategy to be applied to more glycosyltransferases, further expanding the enzymatic toolbox. Furthermore, in vivo biotinylation allows the one-step immobilization and purification of enzymes on streptavidin-functionalized particles, which requires fewer resources and time than protocols with lengthier purification and concentration steps. As seen in Fig. 2d–f, BirA appears as the only measurable contaminant, coeluting with our enzymes. Previous work has identified that the only endogenous protein biotinylated by *E. coli* is acetyl coenzyme A carboxylase (AccB), which is involved in fatty acid biosynthesis^48,49^. This protein is not soluble in *E. coli*^50^, but even if coimmobilized with the Avi-tagged enzymes in SUGAR-TARGET, AccB should not have activity that would negatively impact the glycoprotein. Finally, a clear advantage of our system is that it also takes advantage of high expression yields in microbial hosts, which reduces overall process costs. Future work involves method optimization to allow quantification of the enzyme concentration and immobilization efficiency as well as the identification of key parameters for optimum yields^25,51^.

We subsequently applied our SUGAR-TARGET platform on free glycans, on an mFc produced in glycoengineered *P. pastoris* and on saposin B produced in glycoengineered HEK293 cells. Our aim was to demonstrate a proof-of-concept glycoengineering approach capable of generating human-like glycans on proteins from different sources, whether mammalian or microbial. Furthermore, we showed that extensive host engineering is not required because SUGAR-TARGET can be used as a readily available platform to complement cell-based production and modify a range of proteins. Each step in the cascade approached completion with >95% conversion, with minimal protein losses after each step, highlighting the capacity of our system to yield homogeneous products and glycan intermediates that are otherwise difficult to obtain. Moreover, we were able to generate an alternative highly homogeneous glycoform by modifying the pathway to GnTI–GalT, demonstrating the modularity of the system and the ability to produce an array of bespoke products. We anticipate that SUGAR-TARGET and the spatiotemporal separation it provides can minimize undesired enzyme cross-reactivity often observed in in vitro glycosylation reactions^22^ as well as address enzyme competitive inhibition. This is a limitation often observed in one-pot glycosylation reactions, requiring intermediate purification steps, which increase the cost and complicate scalability^22^. Finally, from a product quality perspective, the therapeutic protein purity depends on the steps before the enzymatic treatment and is not affected by treatment with SUGAR-TARGET. There is no detectable enzyme leaching from the streptavidin beads because of the site-specific, irreversible, strong non-covalent immobilization provided by the biotin–streptavidin system^27,52^.

We also demonstrated the ability of our in-house immobilized GalT to modify the galactosylation profile of three full-length IgGs. Driving galactosylation of IgGs in vitro has been demonstrated on multiple occasions, highlighting the importance of such an endeavor^39,53–55^. In contrast to previous work, our strategy enabled enzyme reusability for four cycles, demonstrating activity over a time span of more than 80 h. This result shows the potential for implementation of SUGAR-TARGET in large-scale industrial processes, including for the continuous modification of glycoproteins.

Based on our IgG galactosylation results, we explored the substrate preferences of our immobilized GalT to further understand the order of galactose addition as well as the distribution of structures (G1 and G2) observed following in vitro treatment. Our results showed that human GalT can modify any substrate with no clear preference for fucosylated or non-fucosylated and agalactosylated or monogalactosylated structures. The 1,6-arm specificity observed here agrees with the existing literature^42^. However, reactions on free glycans appear faster than reactions on glycoproteins, such as IgG produced in CHO cells. Reactions involving glycoproteins produced in cells involve multiple glycan structures (substrates) present at different concentrations, which could explain the heterogeneity observed with the full-length IgGs. Differences in reactivity between free glycans and glycoproteins could also result from steric hindrance from the protein backbone. We anticipate that the reaction conditions can be optimized to further increase activity and homogeneity.

Our work demonstrates notable achievements in the field of in vitro glycosylation. SUGAR-TARGET can be used as a reliable postexpression platform for modification of N-linked glycans of proteins, and it can complement the natural process of glycosylation to produce homogeneous profiles. With the expansion of the enzyme toolbox of SUGAR-TARGET, it is possible to synthesize bespoke mammalian N-linked glycosylation pathways or pathways for other important glycans, such as polysaccharide antigens for vaccine development. Furthermore, our system can theoretically modify any glycoprotein derived from a cell-based or cell-free protein synthesis system, thus allowing for a continuous production-modification bioprocess. Here, our work included material from a yeast system as well as four mammalian systems, whether recombinant (CHO h-IgG and saposin B produced in HEK) or isolated from natural sources (h-IgG and r-lgG). The foundation of SUGAR-TARGET (enzyme immobilization) helps bypass limitations associated with the immobilization of the substrate protein and sequential addition to non-immobilized enzyme pools^56,57^. Although homogeneously glycosylated IgGs, which natively bind with high affinity to protein A through the Fc domain, can be produced by immobilizing the substrate, tailoring is needed for non-IgG substrates. The latter can lead to structural changes to the protein of interest caused by the engineering requirements for immobilization. Furthermore, immobilizing the substrate introduces a number of intermediate processing steps, which are expected to hinder broad industrial adoption and scale-up. By contrast, SUGAR-TARGET is substrate independent and ensures a simple route for enzyme reusability. The latter enables the large-scale application of SUGAR-TARGET, as seen in various industrial setups where immobilized catalysts are routinely used^43^. Finally, the structures produced in vivo and by SUGAR-TARGET are chemically identical, and thus it is reasonable to conclude that glycoproteins modified using our system will behave identically to those produced in cells or even be improved where a specific glycoform is targeted to improve function.

Despite the achievements of our work, there are certain limitations that require careful consideration and further development. Although one-step immobilization/purification, enzyme reusability and use of non-mammalian expression hosts can vastly lower costs, the use of enzymes for in vitro glycosylation reactions can be costly due to the use of nucleotide sugar donors as cosubstrates and solid supports. An in-depth technoeconomic analysis will help evaluate the performance of our platform and the potential cost reduction achievable through, for example, the application of well-established platforms for the enzymatic regeneration of nucleotide sugar donors^58,59^. An area for future development is the inclusion of complex enzymes that perform repeated additions, for example, poly-sialylation, poly-*N*-acetyllactosamine and yeast oligomannose structures of defined size. In such an environment, the optimization of reaction conditions is essential to avoid unwanted intermediates. Finally, as shown in the case of ManII, some enzymes are difficult to produce in economical microbial cell hosts. Although BirA/AviTag can also be used in mammalian hosts, different expression approaches can also be explored. These can include the rational redesign of membrane-bound enzymes to produce soluble catalysts^60^ or the use of novel non-mammalian enzymes^61^.

We anticipate that future work and optimization will enable multiple applications of SUGAR-TARGET. The advancements presented here can serve as a model for both large-scale industrial processes of human-like glycoproteins and the small-scale, on-demand production of bespoke, personalized therapeutics in an economical way, taking advantage of high-yield microbial hosts and enzyme reusability. Furthermore, because our system is modular and easy to use, we envision SUGAR-TARGET serving as a research tool used to synthesize and test the biological roles of different glycans by changing the chosen enzyme pathway.

## Methods

### Growth conditions

*E. coli* strains were cultured in Luria Broth Base (Miller’s LB Broth Base; LB) medium (1% peptone from casein, 0.5% yeast extract and 1% NaCl) at 37 °C. Antibiotics were supplemented for selection (ampicillin, 100 μg ml^–1^; kanamycin, 50 μg ml^–1^; chloramphenicol, 10 μg ml^–1^). Expression was performed in LB medium supplemented with 20% sterile glucose and the appropriate antibiotics. For the generation of pPICZα A mFC, *E. coli* strains were grown in low-salt LB medium (0.5% NaCl) supplemented with 10 μg ml^–1^ zeocin. LB agar (Miller; agar 15 g liter^–1^, tryptone 10 g liter^–1^, NaCl 10 g liter^–1^ and yeast extract 5 g liter^–1^) was used for bacterial growth on agar plates at 37 °C. The antibiotic concentrations used were the same as those used for liquid cultures.

### Strains and plasmids

*E.* *coli* strain NEB 5-α (New England Biolabs) was used for the generation of genetic constructs. The DNA sequence encoding Δ29GnTI from *N. tabacum* (Genbank accession number Y16832) was codon optimized for expression in *E. coli*, chemically synthesized (GeneArt Gene synthesis, Thermo Fisher Scientific) and cloned into the NdeI/EcoRI sites of pMAL-c5x (New England Biolabs) to obtain the plasmid pMAL-c5x-NtGnTI (GnTI). Oligonucleotides encoding the AviTag peptide sequence GLNDIFEAQKIEWHE^26^ with an additional glycine–serine linker in the N terminus were chemically synthesized (Invitrogen, by Thermo Fisher Scientific) and annealed. Subsequently, these were cloned into the C terminus (EcoRI/HindIII sites) of the plasmid, generating the pMAL-c5x-NtGnTI-GS-AviTag plasmid. The DNA sequence encoding human Δ128GalT cDNA was codon optimized for expression in *E. coli*, chemically synthesized (DNA 2.0, Atum) and cloned in the same way as described above, generating the pMAL-c5x-hGalT-GS-AviTag (GalT) plasmid. The DNA encoding *H. cetorum* Δ42SiaT (C-terminal truncation^31^) was codon optimized for expression in *E. coli*, chemically synthesized (GeneArt synthesis, Invitrogen) and cloned using Gibson assembly, generating the pMAL-c5x-HCSiat-GS-AviTag (SiaT) plasmid. DNA sequences for the proteins used in this study are included in the Supplementary Information.

The gene encoding the mFc^36^ was cloned using Gibson assembly into pPICZαA (Thermo Fisher Scientific) with a C-terminal His_6_tag, generating the pPICZαA -mFc-His_6_ plasmid. Finally, the *E. coli* strain AVB101 (Avidity) was used for the isolation of pBirAcm, an IPTG-inducible plasmid containing the gene encoding BirA.

### Expression and biotinylation of glycosyltransferases

For the expression of GnTI, GalT and SiaT, *E. coli* strain Origami 2 DE3 (Novagen) was cotransformed by electroporation with pBirAcm and pMAL-c5x-NtGnTI-GS-AviTag, pMAL-c5x-hGalT-GS-AviTag or pMAL-c5x-HCSiat-GS-AviTag. A single colony of transformed cells was inoculated into 5 ml of LB medium (1% peptone from casein, 0.5% yeast extract and 1% NaCl) containing 100 µg ml^–1^ ampicillin and 10 µg ml^–1^ chloramphenicol and incubated overnight at 37 °C. One liter of LB containing 20% sterile glucose was inoculated with a 1:100 dilution of the starter culture and placed in a shaking incubator at 37 °C until the optical density at 600 nm was 0.6–0.8. Protein expression was induced by the addition of d-biotin (final concentration of 20 µM) and IPTG (final concentration of 0.1 mM) and incubated overnight at 20 °C. Cells were collected by centrifugation (4,000*g*, 30 min), resuspended in lysis buffer (5 ml per gram of cells, 20 mM Tris-HCl (pH 7.5), 200 mM NaCl, 5% glycerol and 0.1 mM phenylmethylsulfonyl fluoride) and sonicated for 5 min with 10-s on/off pulses. The lysate was centrifuged (12,000*g*, 30 min, 4 °C), and the supernatant was filtered using 0.2-μm filters and buffer exchanged in storage buffer (20 mM Tris-HCl (pH 7.4), 200 mM NaCl and 5% glycerol) using PD-10 desalting columns (GE Healthcare). The desalted sample was then aliquoted and stored at −80 °C until the one-step purification/immobilization.

ManII was expressed and purified in the labs of D. Rose and D. Kuntz (University of Waterloo), as previously described^29^. A Lightning-Link Rapid Biotin Type B Labeling Kit (Expedeon) was used for the chemical biotinylation of ManII. The steps followed were as described by the manufacturer.

### Expression and purification of monomeric antibody fragment

The *P. pastoris* (syn. *Komagataella phaffi*) strain GlycoSwitch SuperMan5 (*his4*^–^) was used for the expression of mFc. Expression was performed in 1-liter glass baffled flasks in 100 ml of buffered glycerol/methanol complex medium (BMGY/BMMY; 1% yeast extract, 2% peptone, 100 mM potassium phosphate (pH 6.0), 1.34% yeast nitrogen base, 4 × 10^−5^% d-biotin, 1% glycerol or 0.5% methanol). Cells were initially incubated at 30 °C and 216 r.p.m. for 24 h in BMGY to allow growth. Cells were collected by centrifugation (4,000*g*, 5 min) and resuspended in BMMY to induce expression. Cultures were left to express at 20 °C and 216 r.p.m. for 72 h before being collected (3,000*g*, 15 min, 4 °C). The culture supernatant was immediately used for purification.

To prevent any protein degradation, all purification steps were performed at 4 °C. The supernatant from the *P. pastoris* SuperMan5 cultures was collected, and the pH was adjusted to 9 by using 5 N KOH to precipitate salts. One Pierce protease inhibitor tablet (Thermo Fisher Scientific) cocktail per 50 ml was then added, followed by centrifugation (3,000*g*, 15 min, 4 °C). Clarified supernatants were filtered using 0.2-µm filters, transferred to new 50-ml Falcon tubes containing Ni-NTA resin equilibrated with lysis buffer (20 mM Tris-HCl (pH 9), 10 mM imidazole, 200 mM NaCl and 5% glycerol) and agitated for 1 h to allow protein binding. The resin was recovered by centrifugation (700*g*, 5 min, 4 °C), transferred to a gravity column (Pierce Disposable Columns, 10 ml) and washed twice (five times bed volume) with wash buffer (20 mM Tris-HCl (pH 9), 200 mM NaCl, 5% glycerol and 20 mM imidazole). Finally, mFc was eluted with 5 ml of elution buffer (20 mM Tris-HCl (pH 9), 200 mM NaCl, 250 mM imidazole and 5% glycerol) and buffer exchanged using Amicon Ultra 15 10-kDa filter units (Merck Millipore) in storage buffer (20 mM Tris-HCl (pH 7.5), 200 mM NaCl and 5% glycerol).

### Expression and purification of humanized monoclonal IgG produced in CHO cells

CHO-T127 cells (kindly donated by MedImmune) producing an IgG1 antibody were used for this study. The cells were maintained in CD-CHO medium (Life Technologies) at 36.5 °C ± 0.5 °C at 150 r.p.m. and 5% CO_2_ and were passaged every 3–4 d at a seeding density of 3 × 10^5^ cells per ml. l-Methionine sulfoximine (50 μΜ; Cambridge Biosciences) was supplemented on cell revival and on the first passage. The cells were cultured in 50 ml working volume in 250-ml Erlenmeyer flasks (Corning). On the fourth day of the third passage, 10 ml of the cell culture was centrifuged at 100*g* for 5 min at 25 °C, and the supernatant was isolated.

For purification, the Amicon Pro-Affinity Concentration kit Protein A with 100-kDa Amicon Ultra-0.5 device (Merck Millipore) was used as described by the manufacturer. The storage buffer was 20 mM Tris-HCl (pH 7.4), 200 mM NaCl and 5% glycerol.

### SDS–PAGE analysis and western blotting

SDS–PAGE was performed with MiniPROTEAN Tetra vertical electrophoresis cells (Bio-Rad) using miniPROTEAN TGX precast gels (Bio-Rad). Analysis was performed under reducing conditions, where DTT was added to the sample loading buffer (5×: 0.225 M Tris-HCl (pH 6.8), 50% glycerol, 5% SDS, 0.05% bromophenol blue and 0.25 M DTT) and heated to 95 °C for 10 min. Thermo Scientific PageRuler prestained protein ladder (10–180 kDa) was used for the identification of the band sizes. Gels were stained using SimplyBlue Safe Stain (Thermo Fisher Scientific) and imaged using the NuGenius gel imager (Syngene).

Western blotting was performed using a semidry blotter and anti-His (Biolegend). A BCIP/NBT kit (Thermo Fisher Scientific) was used for band development.

### Reproducibility of gels and western blots

All gel and western blotting experiments were repeated under the described conditions a minimum of three times.

### Confirmation of in vivo biotinylation and densitometry

The gel shift assay was applied as previously described^27^ with one modification: all soluble protein content, following sonication and centrifugation, was used rather than a purified protein. To measure the percent biotinylation of proteins in the gel shift assay, the intensity of the corresponding bands was measured using TOTALLAB CLIQS 1D Gel Image Analysis software. Background subtraction was performed using the rolling ball method.

### Enzyme immobilization

For enzyme immobilization, streptavidin silica particles (1% (wt/vol) 1.0–1.4 µm (Spherotech)) or Dynabeads C1 streptavidin-coated magnetic beads (1% (wt/vol); Thermo Fisher) were used under the following conditions. Bead storage buffer was removed either by centrifugation (5 min at 5,000*g* for silica beads) or with the use of a magnet. The particles were subsequently washed three times with 0.1 M Tris-HCl (pH 7.5) by resuspending and centrifuging or magnetic separation. Immobilization for silica particles was performed using the following protocol. Desalted enzyme sample was mixed with the washed and pelleted particles. A volumetric ratio of 1:2 sample:particles was used. The samples were then diluted with 0.1 M Tris-HCl (pH 7.5) at a final immobilization volume of 1 ml and incubated in a rotary shaker for 1 h at 4 °C. To scale up experiments, larger volumes of samples and particles were used while keeping the 1:2 ratio. Following immobilization, the particles were collected either by centrifugation (3,000*g*, 10 min) or magnetic separation. The pelleted particles were resuspended in storage buffer (20 mM Tris (pH 7.5), 200 mM NaCl and 5% glycerol) and washed three times. For immobilization using magnetic beads, the same steps were followed in a volumetric ratio of 1:0.4, with the final immobilization volume at 250 µl.

### Confirmation of immobilized enzyme activity

Activity of the immobilized enzymes was confirmed by MALDI-TOF MS/MS analysis before the enzyme cascade. Briefly, the activity assay for immobilized GnTI consisted of 0.5 µM M5 glycan (Sigma-Aldrich), 2.5 mM UDP-GlcNAc, 100 mM MES (pH 6.5) and 1 mM MnCl_2_ and was incubated overnight at 25 °C with continuous shaking. The activity assay for immobilized ManII consisted of 0.1 mM ZnSO_4_, 50 mM MES (pH 5.6) and 0.4 μM substrate from the reaction of immobilized GnTI and was incubated overnight at 37 °C with continuous shaking. The activity assay for immobilized GalT consisted of 16 μM GlcNAc, 0.16 mM UDP-Gal (Merck), 16 mM GlcNAc (Sigma-Aldrich), 25 mM Tris-HCl (pH 7.5) and 10 mM MnCl_2_ and was incubated overnight at 37 °C with continuous shaking. Finally, the activity assay for immobilized SiaT consisted of 0.8 μM G2F in 35 mM MES (pH 6.5), 2 mM CMP-NANA, 0.1% Triton X-100 and 10 mM MgCl_2_ and was incubated overnight at 37 °C with continuous shaking.

### Immobilized enzyme cascade

The following protocol was used for the cascade on free glycans. M5 glycan (0.5 µM) was treated with immobilized GnTI in the enzyme reaction buffer (10 mM UDP-GlcNAc, 100 mM MES (pH 6.5) and 1 mM MnCl_2_) and was incubated overnight at 25 °C with continuous shaking. The immobilized enzyme was then removed by centrifugation (5 min, 5,000*g*), and a small sample (0.1 µM glycan) was collected for subsequent MALDI-TOF MS analysis. The remaining reaction (0.4 µM glycan) was treated with immobilized ManII in the enzyme reaction buffer (0.1 mM ZnSO_4_ and 50 mM MES (pH 5.6)) and was incubated overnight at 37 °C with continuous shaking. The immobilized enzyme was removed by centrifugation (5 min, 5,000*g*), and a small sample (0.1 µM glycan) was collected for subsequent MALDI-TOF MS analysis. The remaining reaction was treated with immobilized GalT in the enzyme reaction buffer (6 mM UDP-Gal, 100 mM Tris-HCl (pH 7.5) and 10 mM MnCl_2_) and was incubated overnight at 37 °C with continuous shaking. The enzyme was removed by centrifugation (5 min, 5,000*g*) and analyzed by MALDI-TOF MS. For the final step, the remaining reaction was treated with immobilized SiaT in the enzyme reaction buffer (35 mM MES (pH 6.5), 2 mm CMP-NANA, 0.1% Triton X-100 and 10 mM MgCl_2_) and incubated overnight at 37 °C with continuous shaking. The enzyme was removed by centrifugation (5 min, 5,000*g*) and analyzed by MALDI-TOF MS.

The cascade on mFc was performed as described for free glycans with the following modifications: (1) the enzymes were immobilized on magnetic beads to facilitate sampling, (2) 100 µg of mFc was used instead of free glycans, (3) 25 µg of treated mFc was removed at the end of each reaction for MALDI-TOF MS analysis, and (4) following completion of the SiaT reaction and removal of the enzyme, mFc was buffer exchanged to a buffer suitable for MALDI-TOF MS analysis (here, 20 mM Tris-HCl (pH 7.5) and 200 mM NaCl).

### IgG galactosylation reactions

For the galactosylation experiments, GalT was immobilized on magnetic streptavidin beads. To ensure maximum enzyme concentration, the immobilization experiment was scaled up fourfold. The antibodies used were (1) purified CHO h-IgG, (2) h-IgG (Sigma-Aldrich; resuspended in deionized water and stored at 4 °C) and (3) r-IgG (Sigma-Aldrich; resuspended in deionized water and stored at 4 °C). The reaction consisted of the following components: 110 µg of IgG (CHO h-IgG/h-IgG/r-IgG), 20 mM MnCl_2_, 20 mM Tris-HCl (pH 7.5) and 6 mM UDP-Gal. The reaction took place overnight at 37 °C on a shaking platform. After the end of the reactions, the immobilized enzyme was removed with a magnet, and the supernatant containing the IgGs was concentrated using Vivaspin 500 ultracentrifugation spin columns (100 kDa) and used for CE analysis.

For the reusability experiments, only CHO h-IgG was used. Following completion of the overnight reaction of immobilized GalT and CHO h-IgG, the immobilized enzyme was recovered. The beads were washed three times in protein storage buffer (20 mM Tris-HCl (pH 7.5), 200 mM NaCl and 5% glycerol) and were subsequently used for a new reaction as described earlier. At the end of each reaction and after removing the immobilized enzymes, the supernatant containing CHO h-IgG was removed, concentrated and used for CE analysis.

### Galactosylation of different substrates

For the galactosylation experiments, GalT was immobilized on magnetic streptavidin beads. The substrates used were G0 and G0F (Dextra Laboratories), 0.8 μM free glycan, 10 mM MnCl_2_, 20 mM Tris-HCl (pH 7.5) and 6 mM UDP-Gal. Following completion of the reactions, the samples were stored at −80 °C until preparation for CE analysis. For the analysis, the area of each peak was normalized against the area of the internal standard DP15.

### Matrix-assisted laser desorption/ionization-time-of-flight mass spectrometry analysis

Free glycans were lyophilized overnight. Permethylation was performed using a sodium hydroxide method, as described previously^62^. Permethylated samples were combined in a 1:3 ratio with 10 mg ml^–1^ 3,4-diaminobenzophenone (Acros Organics) matrix in 75% acetonitrile. The analysis was performed in reflector positive ion mode using a 4800 MALDI-TOF/TOF (Applied Biosystems) mass spectrometer. MS spectra were assigned and annotated with the help of GlycoWorkbench (version 2.1) software^63^.

For the glycosylation analysis of mFc, the methods used were similar to those described for the free glycans with the following addition: glycans were first removed and purified using the PA 800 Plus Fast Glycan workflow (AB Sciex). The labeling step was not performed for MALDI-TOF MS analysis.

### Capillary electrophoresis

For CE, the C1000HT platform (AB Sciex) was used. The SCIEX C100HT Glycan Labeling and Analysis kit was used for sample preparations, and the steps followed were as described by the manufacturer. Analysis of the CE separation results to identify glycan structures in the samples followed by review of the results were performed with C100HT built-in 32Karat software (version 10.1.11).

### Reporting summary

Further information on research design is available in the Nature Portfolio Reporting Summary linked to this article.

## Online content

Any methods, additional references, Nature Portfolio reporting summaries, source data, extended data, supplementary information, acknowledgements, peer review information; details of author contributions and competing interests; and statements of data and code availability are available at 10.1038/s41589-023-01539-4.

### Supplementary Information

Supplementary Information Supplementary Fig. 1 and Notes 1–3.

Reporting Summary

### Source data

Source Data Fig. 2 Unprocessed gel for Fig. 2b–f.

Source Data Fig. 3 Unprocessed gel for Fig. 3c.

Source Data Figs. 4 and 5 Statistical source data.

Source Data Fig. 4 Electropherograms of CE data for Fig. 4b–d.

Source Data Fig. 6 Electropherograms of CE data.

Source Data Extended Data Fig. 1 Unprocessed gel.

Source Data Extended Data Fig. 2 Unprocessed gel.

## Data Availability

All data files are deposited as a Mendeley dataset and can be accessed via the following link: 10.17632/mnytyftvmr.1. Source data are provided with this paper.
