# Supplementary material for: Immobilized enzyme cascade for targeted glycosylation
Source: Nat Chem Biol. 2024 Feb 6;20(6):732–41. doi: 10.1038/s41589-023-01539-4 (PMC11142912; doi:10.1038/s41589-023-01539-4)
Supplement: Supplementary file 1 — Supplementary Fig. 1 and Notes 1–3. [file 41589_2023_1539_MOESM1_ESM.pdf]

nature chemical biology

Article https://doi.org/10.1038/s41589-023-01539-4

Immobilized enzyme cascade for targeted glycosylation

In the format provided by the authors and unedited

Supplementary Information

Supplementary Figure 1

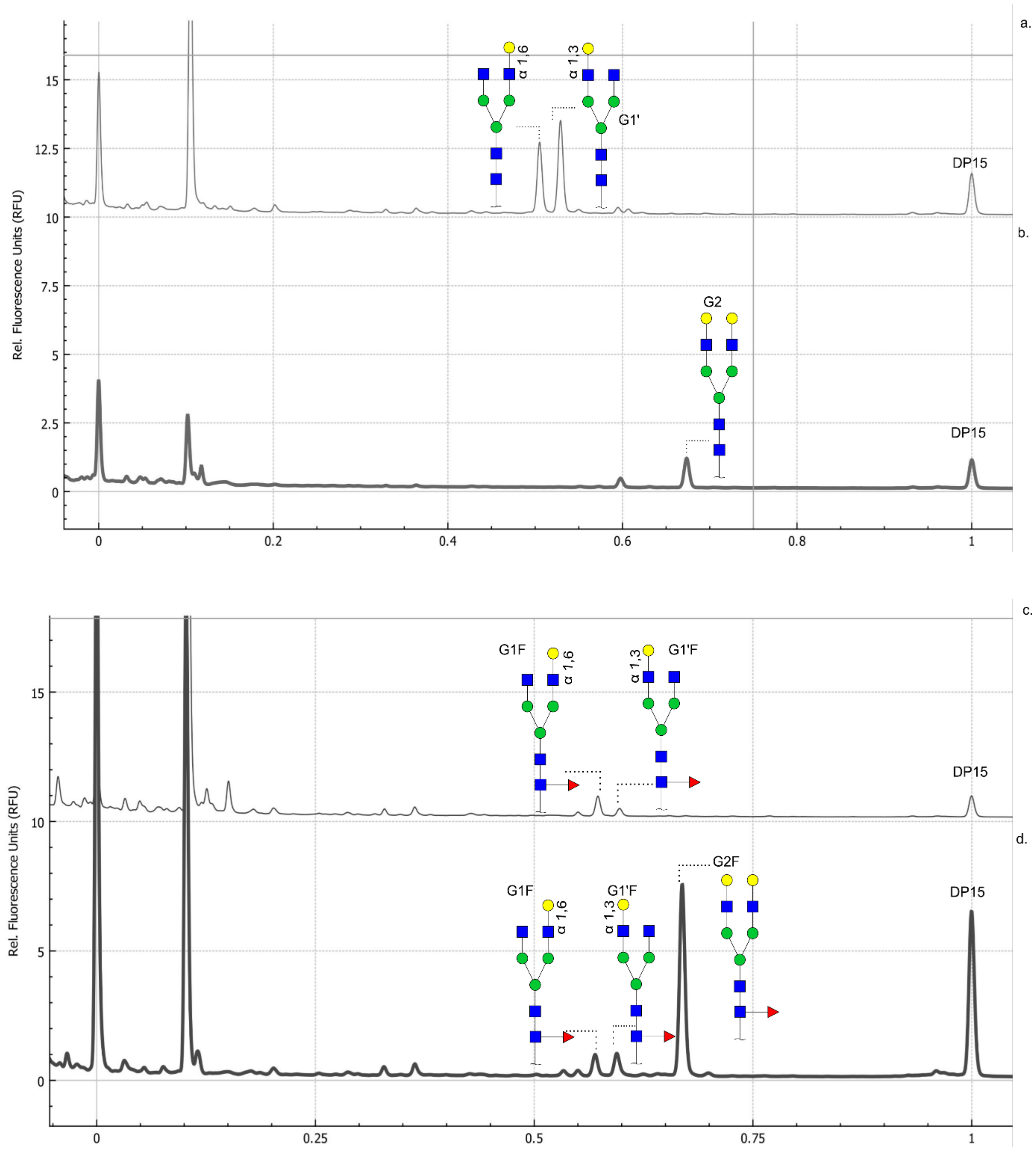

Supplementary Figure 1: Reaction of immobilised GalT with glycans; a. Electropherogram of starting substrate G1 and G1’; b. Electropherogram of reaction products of G1 and G1’ following 15-min treatment with immobilised GalT; c. Electropherogram of starting substrate G1F and G1’F; d. Electropherogram of reaction products of G1F and G1’F following 15-min treatment with immobilised GalT. All areas were normalised against internal standard DP15.

Supplementary note 1.

DNA sequence of *N. tabacum* N-Acetylglucosaminyltransferase I (GnTI) used in this study.

Blue: Maltose Binding Protein (MBP)
Green: GnTI
Red: AviTag
Purple: GS linker

ATGAAAATCGAAGAAGGTAAACTGGTAATCTGGATTAACGGCGATAAAGGCTATAACGGTCTCGCTGAAGTC
GGTAAGAAATTCGAGAAAGATACCGGAATTAAAGTCACCGTTGAGCATCCGGATAAACTGGAAGAGAAATTC
CCACAGGTTGCGGCAACTGGCGATGGCCCTGACATTATCTTCTGGGCACACGACCGCTTTGGTGGCTACGCTC
AATCTGGCCTGTTGGCTGAAATCACCCCGGACAAAGCGTTCCAGGACAAGCTGTATCCGTTTACCTGGGATGC
CGTACGTTACAACGGCAAGCTGATTGCTTACCCGATCGCTGTTGAAGCGTTATCGCTGATTTATAACAAAGATC
TGCTGCCGAACCCGCCAAAAACCTGGGAAGAGATCCCGGCGCTGGATAAAGAACTGAAAGCGAAAGGTAAG
AGCGCGCTGATGTTCAACCTGCAAGAACCGTACTTCACCTGGCCGCTGATTGCTGCTGACGGGGGTTATGCGT
TCAAGTATGAAAACGGCAAGTACGACATTAAAGACGTGGGCGTGGATAACGCTGGCGCGAAAGCGGGTCTG
ACCTTCCTGGTTGACCTGATTAAAAACAAACACATGAATGCAGACACCGATTACTCCATCGCAGAAGCTGCCTT
TAATAAAGGCGAAACAGCGATGACCATCAACGGCCCGTGGGCATGGTCCAACATCGACACCAGCAAAGTGAA
TTATGGTGTAACGGTACTGCCGACCTTCAAGGGTCAACCATCCAAACCGTTCGTTGGCGTGCTGAGCGCAGGT
ATTAACGCCGCCAGTCCGAACAAAGAGCTGGCAAAAGAGTTCCTCGAAAACTATCTGCTGACTGATGAAGGT
CTGGAAGCGGTTAATAAAGACAAACCGCTGGGTGCCGTAGCGCTGAAGTCTTACGAGGAAGAGTTGGTGAA
AGATCCGCGTATTGCCGCCACTATGGAAAACGCCCAGAAAGGTGAAATCATGCCGAACATCCCGCAGATGTC
CGCTTTCTGGTATGCCGTGCGTACTGCGGTGATCAACGCCGCCAGCGGTCGTCAGACTGTCGATGAAGCCCTG
AAAGACGCGCAGACTAATTCGAGCTCGAACAACAACAACAATAACAATAACAACAACCTCGGGATCGAGGGA
AGGATTTCACAtatgGCGACGCAATCGGAATATGCGGATCGCTTGGCAGCGGCGATTGAAGCCGAAAACCATT
GCACGTCACAGACCCGCTTACTGATCGATCAAATCAGTCAGCAACAAGGACGCATTGTAGCCCTGGAGGAAC
AGATGAAACGTCAGGATCAGGAGTGTCGCCAATTACGTGCTTTGGTTCAGGATCTTGAGTCGAAAGGGATTA
AGAAATTGATCGGCAATGTTCAAATGCCTGTTGCTGCAGTAGTGGTCATGGCCTGCAATCGTGCGGATTACCT
CGAGAAAACCATCAAATCCATCCTGAAGTATCAGATTAGCGTTGCACCGAAATACCCTCTGTTTATCTCCCAAG
ATGGTTCTCATCCGGATGTCCGCAAACTGGCGTTAAGCTACGATCAACTGACCTATATGCAGCATCTGGATTTT
GAACCGGTGCACACTGAACGTCCTGGCGAATTAATCGCGTATTACAAAATTGCACGCCACTACAAATGGGCCC
TTGACCAGCTCTTTTACAAGCACAACTTTAGCCGGGTGATCATTCTTGAGGACGATATGGAAATTGCCCCAGAC
TTCTTCGACTTCTTTGAAGCCGGAGCTACTCTGCTGGATCGCGATAAGTCGATTATGGCGATCAGTAGCTGGA
ACGATAACGGGCAGATGCAGTTTGTGCAAGATCCCTATGCTTTATATCGCTCAGACTTCTTTCCGGGTCTGGGT
TGGATGTTGAGTAAATCGACATGGGACGAACTGAGCCCGAAATGGCCGAAAGCTTACTGGGATGACTGGTTG
CGCCTGAAGGAAAACCATCGTGGTCGTCAGTTCATTCGCCCGGAAGTGTGTCGTAGCTATAACTTTGGTGAAC
ATGGTAGCAGTCTGGGCCAGTTCTTTAAACAGTATCTGGAACCCATCAAACTCAATGACGTCCAGGTCGACTG
GAAATCCATGGATCTTTCTTATCTGCTGGAGGACAATTACGTGAAACACTTTGGCGATCTGGTGAAGAAAGCG
AAACCGATTCATGGTGCCGACGCAGTGCTGAAAGCGTTTAACATTGATGGGGATGTTCGCATTCAGTACCGTG
ATCAGCTGGACTTTGAAGATATTGCACGTCAGTTTGGCATTTTCGAAGAGTGGAAAGATGGCGTACCACGTGC
GGCCTATAAAGGCATCGTAGTGTTCCGCTATCAGACGTCACGCCGGGTTTTCCTCGTCGGCCCAGACTCTCTGC
AGCAACTGGGCAATGAAGATACCgaattCGGTTCTGGTCTTAATGATATTTTTGAAGCTCAGAAGATTGAATGG
CATGAATAA

Supplementary note 2.

DNA sequence of *H. sapiens* β-1,4-Galactosyltransferase (GalT) used in this study.

Blue: Maltose Binding Protein (MBP)
Green: GalT
Red: AviTag
Purple: GS linker

ATGAAAATCGAAGAAGGTAAACTGGTAATCTGGATTAACGGCGATAAAGGCTATAACGGTCTCGCTGAAGTC
GGTAAGAAATTCGAGAAAGATACCGGAATTAAAGTCACCGTTGAGCATCCGGATAAACTGGAAGAGAAATTC
CCACAGGTTGCGGCAACTGGCGATGGCCCTGACATTATCTTCTGGGCACACGACCGCTTTGGTGGCTACGCTC
AATCTGGCCTGTTGGCTGAAATCACCCCGGACAAAGCGTTCCAGGACAAGCTGTATCCGTTTACCTGGGATGC
CGTACGTTACAACGGCAAGCTGATTGCTTACCCGATCGCTGTTGAAGCGTTATCGCTGATTTATAACAAAGATC
TGCTGCCGAACCCGCCAAAAACCTGGGAAGAGATCCCGGCGCTGGATAAAGAACTGAAAGCGAAAGGTAAG
AGCGCGCTGATGTTCAACCTGCAAGAACCGTACTTCACCTGGCCGCTGATTGCTGCTGACGGGGGTTATGCGT
TCAAGTATGAAAACGGCAAGTACGACATTAAAGACGTGGGCGTGGATAACGCTGGCGCGAAAGCGGGTCTG
ACCTTCCTGGTTGACCTGATTAAAAACAAACACATGAATGCAGACACCGATTACTCCATCGCAGAAGCTGCCTT
TAATAAAGGCGAAACAGCGATGACCATCAACGGCCCGTGGGCATGGTCCAACATCGACACCAGCAAAGTGAA
TTATGGTGTAACGGTACTGCCGACCTTCAAGGGTCAACCATCCAAACCGTTCGTTGGCGTGCTGAGCGCAGGT
ATTAACGCCGCCAGTCCGAACAAAGAGCTGGCAAAAGAGTTCCTCGAAAACTATCTGCTGACTGATGAAGGT
CTGGAAGCGGTTAATAAAGACAAACCGCTGGGTGCCGTAGCGCTGAAGTCTTACGAGGAAGAGTTGGTGAA
AGATCCGCGTATTGCCGCCACTATGGAAAACGCCCAGAAAGGTGAAATCATGCCGAACATCCCGCAGATGTC
CGCTTTCTGGTATGCCGTGCGTACTGCGGTGATCAACGCCGCCAGCGGTCGTCAGACTGTCGATGAAGCCCTG
AAAGACGCGCAGACTAATTCGAGCTCGAACAACAACAACAATAACAATAACAACAACCTCGGGATCGAGGGA
AGGATTTCACAtatgGCCTGCCCTGAGGAAAGCCCACTGTTGGTGGGCCCAATGCTGATCGAGTTTAACATGCC
GGTGGACCTGGAACTGGTGGCGAAACAGAACCCGAACGTCAAAATGGGCGGCCGTTACGCACCGCGTGACT
GCGTTAGCCCGCACAAAGTCGCGATCATTATTCCGTTCCGCAATCGCCAAGAGCATCTGAAGTACTGGCTGTA
CTATCTGCATCCAGTTCTGCAACGTCAGCAATTGGACTACGGTATTTACGTTATCAATCAAGCCGGCGACACGA
TCTTTAATCGTGCTAAGTTGCTGAATGTTGGTTTTCAAGAAGCGCTGAAAGACTACGACTACACCTGTTTCGTG
TTCTCCGACGTTGACCTGATTCCGATGAATGATCACAATGCGTACCGCTGTTTTTCTCAGCCGCGTCACATCAG
CGTAGCGATGGATAAGTTTGGTTTCAGCCTGCCGTATGTGCAGTATTTTGGTGGCGTCAGCGCACTGAGCAAG
CAACAGTTTCTCACGATTAACGGTTTCCCGAACAACTATTGGGGTTGGGGTGGCGAAGATGATGATATCTTCA
ACCGTCTGGTGTTCCGTGGTATGAGCATTAGCCGCCCGAACGCTGTGGTTGGCCGTTGCCGTATGATTCGTCA
TAGCCGCGACAAGAAAAATGAACCGAATCCTCAGCGTTTCGATCGTATCGCACACACCAAAGAAACTATGTTG
AGCGACGGCTTAAACAGCCTGACCTATCAAGTCTTGGATGTTCAACGCTATCCGCTGTACACGCAGATTACCG
TGGACATTGGCACCCCGAGCGaattCGGTTCTGGTCTTAATGATATTTTTGAAGCTCAGAAGATTGAATGGCAT
GAATAA

Supplementary note 3.

DNA sequence of *H. cetorum* β-galactoside α-2,6-sialyltransferase I (SiaT) used in this study.

Blue: Maltose Binding Protein (MBP)
Green: SiaT
Red: AviTag
Purple: GS linker

ATGAAAATCGAAGAAGGTAAACTGGTAATCTGGATTAACGGCGATAAAGGCTATAACGGTCTCGCTGAAGTC
GGTAAGAAATTCGAGAAAGATACCGGAATTAAAGTCACCGTTGAGCATCCGGATAAACTGGAAGAGAAATTC
CCACAGGTTGCGGCAACTGGCGATGGCCCTGACATTATCTTCTGGGCACACGACCGCTTTGGTGGCTACGCTC
AATCTGGCCTGTTGGCTGAAATCACCCCGGACAAAGCGTTCCAGGACAAGCTGTATCCGTTTACCTGGGATGC
CGTACGTTACAACGGCAAGCTGATTGCTTACCCGATCGCTGTTGAAGCGTTATCGCTGATTTATAACAAAGATC
TGCTGCCGAACCCGCCAAAAACCTGGGAAGAGATCCCGGCGCTGGATAAAGAACTGAAAGCGAAAGGTAAG
AGCGCGCTGATGTTCAACCTGCAAGAACCGTACTTCACCTGGCCGCTGATTGCTGCTGACGGGGGTTATGCGT
TCAAGTATGAAAACGGCAAGTACGACATTAAAGACGTGGGCGTGGATAACGCTGGCGCGAAAGCGGGTCTG
ACCTTCCTGGTTGACCTGATTAAAAACAAACACATGAATGCAGACACCGATTACTCCATCGCAGAAGCTGCCTT
TAATAAAGGCGAAACAGCGATGACCATCAACGGCCCGTGGGCATGGTCCAACATCGACACCAGCAAAGTGAA
TTATGGTGTAACGGTACTGCCGACCTTCAAGGGTCAACCATCCAAACCGTTCGTTGGCGTGCTGAGCGCAGGT
ATTAACGCCGCCAGTCCGAACAAAGAGCTGGCAAAAGAGTTCCTCGAAAACTATCTGCTGACTGATGAAGGT
CTGGAAGCGGTTAATAAAGACAAACCGCTGGGTGCCGTAGCGCTGAAGTCTTACGAGGAAGAGTTGGTGAA
AGATCCGCGTATTGCCGCCACTATGGAAAACGCCCAGAAAGGTGAAATCATGCCGAACATCCCGCAGATGTC
CGCTTTCTGGTATGCCGTGCGTACTGCGGTGATCAACGCCGCCAGCGGTCGTCAGACTGTCGATGAAGCCCTG
AAAGACGCGCAGACTAATTCGAGCTCGAACAACAACAACAATAACAATAACAACAACCTCGGGATCGAGGGA
AGGATTTCACATATGAGCGAGAAAATCTTTAGCCAGGTGGATGAGAAAAATCAGAAAAAGCCGCTGATTATT
GCAGGTAATGGTCCGAGCATTAAAGATCTGGATTATAGCCTGTTTCCGAAAGATTTTGATGTGTTTCGTTGCA
ACCAGTTCTATTTCGAGGATAAATACTATCTGGGCAAAGAGGTTAAAGGCGTTTTCTTTAATCCGTGCGTGTTT
CATAATCAGATGAACACCGCAAAACACCTGATCGATAACAACGAGTATTATATCGAACAGTTCTTCTGCAGCG
TGTCCAAAGAACAGCATGATTTTAATGGTGATTACCAGACCATTCTGAGCGTGGATGAATATCTGCGTGCAAA
TTATCCGTTTGTGCGTGATACCTTTAGTCTGTTTGGTGAACATGAAGAAATCCTGAACCACGTGAAATATCACC
TGAAAACCTATAGCAAAGAACTGAGTGCCGGTGTTCTGATGCTGCTGAGCGCAATTGTTCTGGGTTATAAAGA
AATTTATCTGGTCGGTGTTGATTTCGGTGCAAATAGCTGGGGTCATTTCTATGATGATAATCAGAGCCAGCACT
TCATTAATCATATGGCCGATTGCCACAACATCTATTATGATATGCTGACCATTTACCTGTGCCAGAAATATGCA
AAACTGTATGCACTGGTTCCGAATAGTCCGCTGAATCATCTGCTGCCGCTGAACCTGCAGGCAAATCATGTTTT
TGAACTGCTGGATAAACCGATTGGTTATACCAGCGATCTGATTGTTAGCAGTCCGCTGGAAGAAAAACTGCTT
GAAAGCAAAAACATCGATGAAGAATTCGGTTCTGGTCTTAATGATATTTTTGAAGCTCAGAAGATTGAATGGC
ATGAATAA
