# Supplementary material for: Immobilized enzyme cascade for targeted glycosylation
Source: Nat Chem Biol. 2024 Feb 6;20(6):732–41. doi: 10.1038/s41589-023-01539-4 (PMC11142912; doi:10.1038/s41589-023-01539-4)
Supplement: Supplementary file 2 — Reporting Summary [file 41589_2023_1539_MOESM2_ESM.pdf]

nature portfolio

Corresponding author(s): Cleo Kontoravdi, Karen Polizzi

Last updated by author(s): Dec 6, 2023

Reporting Summary

Nature Portfolio wishes to improve the reproducibility of the work that we publish. This form provides structure for consistency and transparency in reporting. For further information on Nature Portfolio policies, see our Editorial Policies and the Editorial Policy Checklist.

Statistics

For all statistical analyses, confirm that the following items are present in the figure legend, table legend, main text, or Methods section.

n/a	Confirmed	
☐	☒	The exact sample size (*n*) for each experimental group/condition, given as a discrete number and unit of measurement
☐	☒	A statement on whether measurements were taken from distinct samples or whether the same sample was measured repeatedly
☐	☒	The statistical test(s) used AND whether they are one- or two-sided<br>*Only common tests should be described solely by name; describe more complex techniques in the Methods section.*
☒	☐	A description of all covariates tested
☐	☒	A description of any assumptions or corrections, such as tests of normality and adjustment for multiple comparisons
☐	☒	A full description of the statistical parameters including central tendency (e.g. means) or other basic estimates (e.g. regression coefficient) AND variation (e.g. standard deviation) or associated estimates of uncertainty (e.g. confidence intervals)
☐	☒	For null hypothesis testing, the test statistic (e.g. *F*, *t*, *r*) with confidence intervals, effect sizes, degrees of freedom and *P* value noted<br>*Give P values as exact values whenever suitable.*
☒	☐	For Bayesian analysis, information on the choice of priors and Markov chain Monte Carlo settings
☒	☐	For hierarchical and complex designs, identification of the appropriate level for tests and full reporting of outcomes
☐	☒	Estimates of effect sizes (e.g. Cohen's *d*, Pearson's *r*), indicating how they were calculated

Our web collection on statistics for biologists contains articles on many of the points above.

Software and code

Policy information about availability of computer code

Data collection	Sciex CH100T 32 Karat Operating System (Version 10.1.11)
Data analysis	TOTALLAB CLIQS Gel Image analysis software, GlycoWorkBench 2.1,

For manuscripts utilizing custom algorithms or software that are central to the research but not yet described in published literature, software must be made available to editors and reviewers. We strongly encourage code deposition in a community repository (e.g. GitHub). See the Nature Portfolio guidelines for submitting code & software for further information.

Data

Policy information about availability of data

All manuscripts must include a data availability statement. This statement should provide the following information, where applicable:

- Accession codes, unique identifiers, or web links for publicly available datasets
- A description of any restrictions on data availability
- For clinical datasets or third party data, please ensure that the statement adheres to our policy

All data are available in the manuscript and its supplementary information

Research involving human participants, their data, or biological material

Policy information about studies with human participants or human data. See also policy information about sex, gender (identity/presentation), and sexual orientation and race, ethnicity and racism.

Reporting on sex and gender	No human subjects
Reporting on race, ethnicity, or other socially relevant groupings	No human subjects
Population characteristics	No human subjects
Recruitment	No human subjects
Ethics oversight	No human subjects

Note that full information on the approval of the study protocol must also be provided in the manuscript.

Field-specific reporting

Please select the one below that is the best fit for your research. If you are not sure, read the appropriate sections before making your selection.

☒ Life sciences ☐ Behavioural & social sciences ☐ Ecological, evolutionary & environmental sciences

For a reference copy of the document with all sections, see nature.com/documents/nr-reporting-summary-flat.pdf

Life sciences study design

All studies must disclose on these points even when the disclosure is negative.

Sample size	No sample size calculations were performed. Samples were measured in duplicate to provide information on reproducibility with minimal use of materials
Data exclusions	No data were excluded
Replication	Experiments were performed with multiple enzyme batches over the lifetime of the project. Representative data are shown in the manuscript
Randomization	Randomization is not relevant for this type of study. We are not sampling natural phenomena. Experiments are done in low throughput and so all measurements were made at the same time to avoid introducing temporal effects.
Blinding	Blinding is not relevant. Measurements are quantitative and not subject to bias by the observer.

Reporting for specific materials, systems and methods

We require information from authors about some types of materials, experimental systems and methods used in many studies. Here, indicate whether each material, system or method listed is relevant to your study. If you are not sure if a list item applies to your research, read the appropriate section before selecting a response.

Materials & experimental systems

n/a	Involved in the study	
☐	☒	Antibodies
☐	☒	Eukaryotic cell lines
☒	☐	Palaeontology and archaeology
☒	☐	Animals and other organisms
☒	☐	Clinical data
☒	☐	Dual use research of concern
☒	☐	Plants

Methods

n/a	Involved in the study	
☒	☐	ChIP-seq
☒	☐	Flow cytometry
☒	☐	MRI-based neuroimaging

Antibodies

Antibodies used	We did not employ antibodies as a research tool (e.g. for ELISA or Western blotting). Antibodies were used as model glycoproteins to which the system was applied for modification of the glycans. This work is independent of the antigen target of the antibody

Validation	No validation of antibodies was used since the antibody was used solely as a model protein

Eukaryotic cell lines

Policy information about cell lines and Sex and Gender in Research

Cell line source(s)	HEK293 GntI -/- and CHO T127 cells were used to express model proteins. The CHOT127 were obtained from MedImmune to the Kontoravdi Group under MTA. The HEK293 CntI -/- cells were obtained to the Rose Group under MTA
Authentication	No authentication was undertaken. No measurements were undertaken on the cells themselves during the work. They served only as a host for the expression of proteins used as a starting material for glycan modification.
Mycoplasma contamination	Not tested
Commonly misidentified lines (See ICLAC register)	While some HEK lines are on the register, this particular cell line is not and the knockout makes its phenotype unique and easy to identfy.

Plants

Seed stocks	*Report on the source of all seed stocks or other plant material used. If applicable, state the seed stock centre and catalogue number. If plant specimens were collected from the field, describe the collection location, date and sampling procedures.*
Novel plant genotypes	*Describe the methods by which all novel plant genotypes were produced. This includes those generated by transgenic approaches, gene editing, chemical/radiation-based mutagenesis and hybridization. For transgenic lines, describe the transformation method, the number of independent lines analyzed and the generation upon which experiments were performed. For gene-edited lines, describe the editor used, the endogenous sequence targeted for editing, the targeting guide RNA sequence (if applicable) and how the editor was applied.*
Authentication	*Describe any authentication procedures for each seed stock used or novel genotype generated. Describe any experiments used to assess the effect of a mutation and, where applicable, how potential secondary effects (e.g. second site T-DNA insertions, mosiacism, off-target gene editing) were examined.*
