# Supplementary figures and images for: Immobilized enzyme cascade for targeted glycosylation
Source: Nat Chem Biol. 2024 Feb 6;20(6):732–41. doi: 10.1038/s41589-023-01539-4 (PMC11142912; doi:10.1038/s41589-023-01539-4)

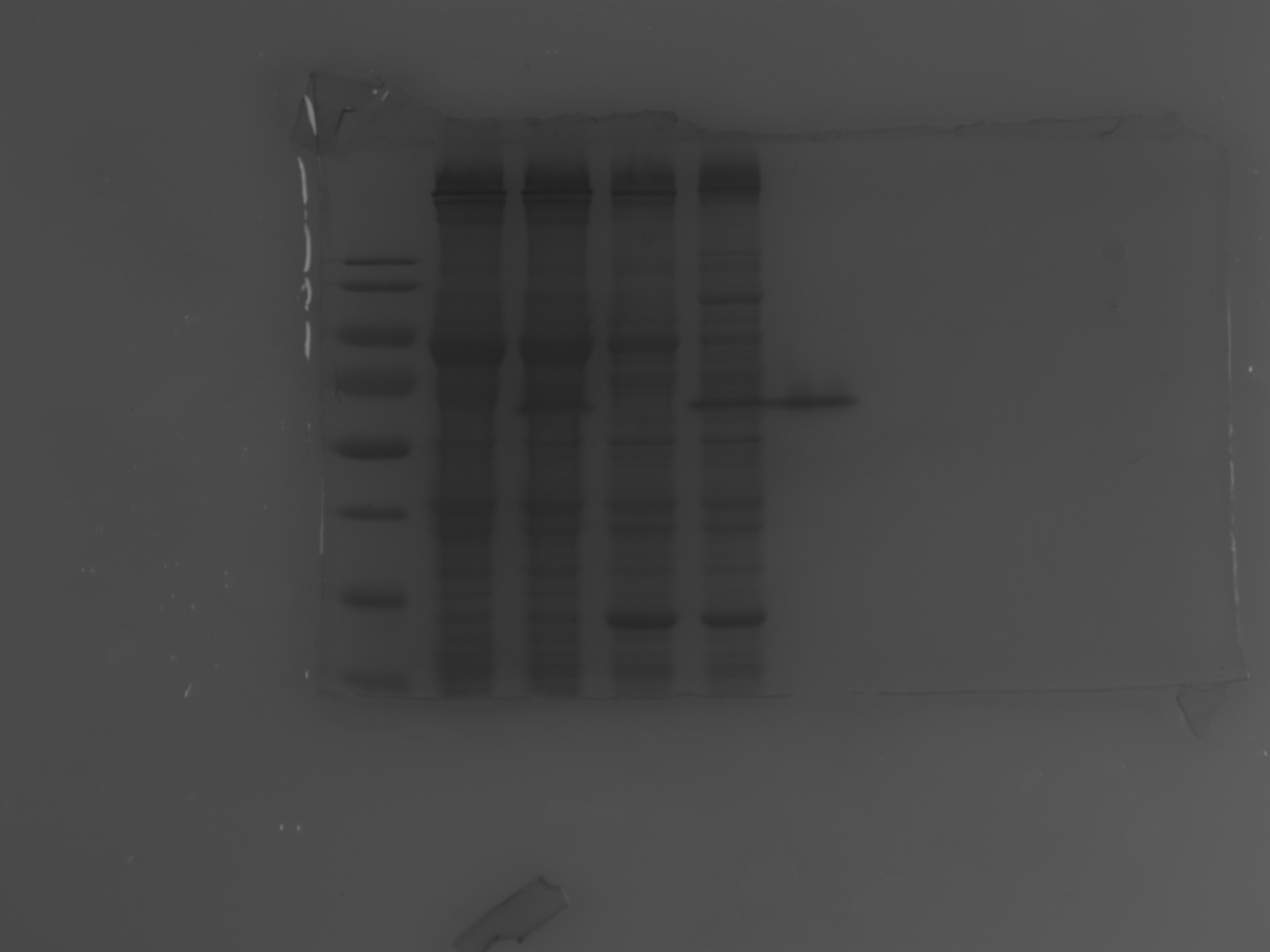

Supplement: Supplementary file 3 — Unprocessed gel for Fig. 2b–f. [file 41589_2023_1539_MOESM3_ESM.zip › Makrydaki_E_SourceDataFig2/Makrydaki_E_SourceDataFig2b.tif]

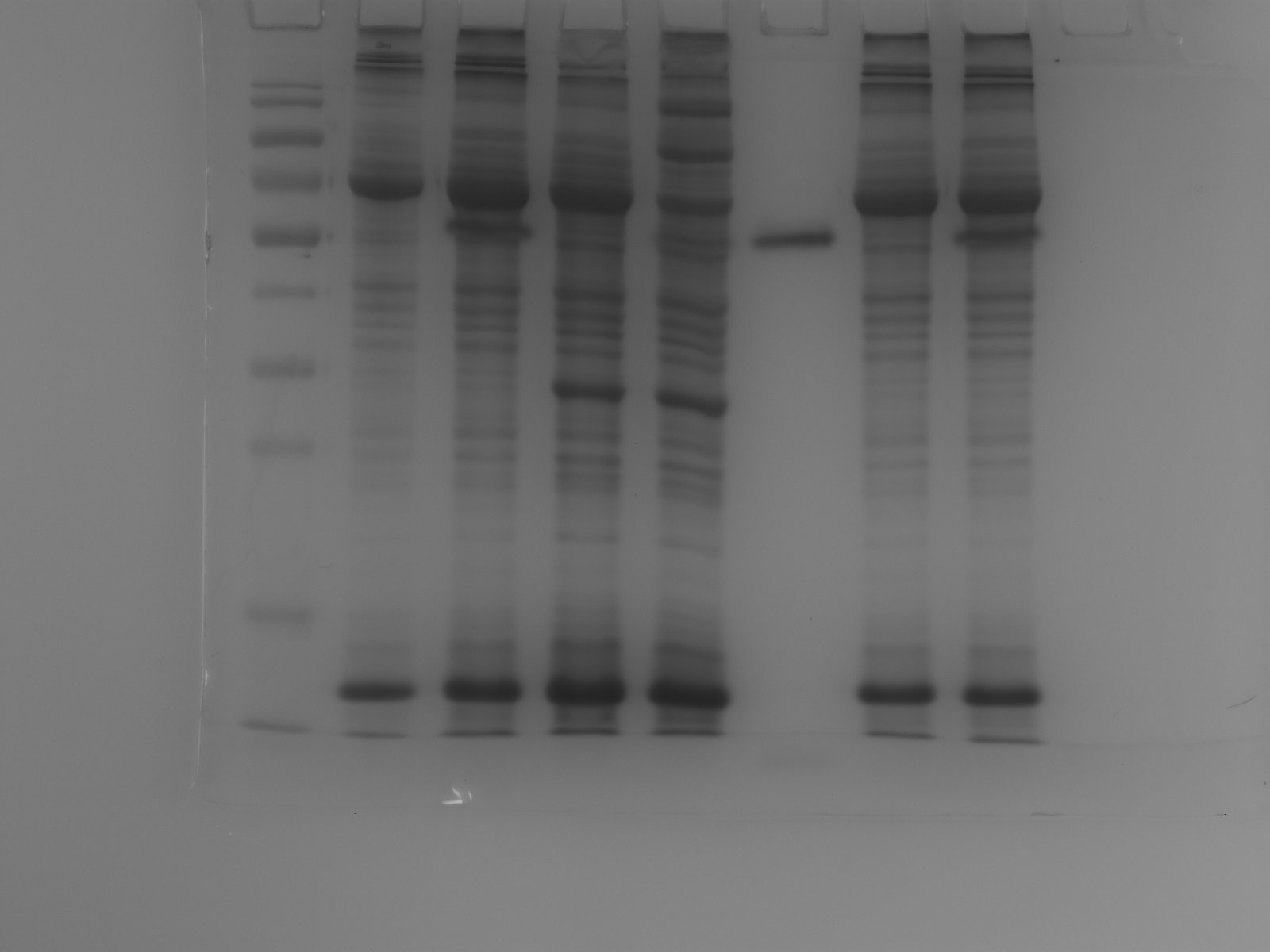

Supplement: Supplementary file 3 — Unprocessed gel for Fig. 2b–f. [file 41589_2023_1539_MOESM3_ESM.zip › Makrydaki_E_SourceDataFig2/Makrydaki_E_SourceDataFig2c.tif]

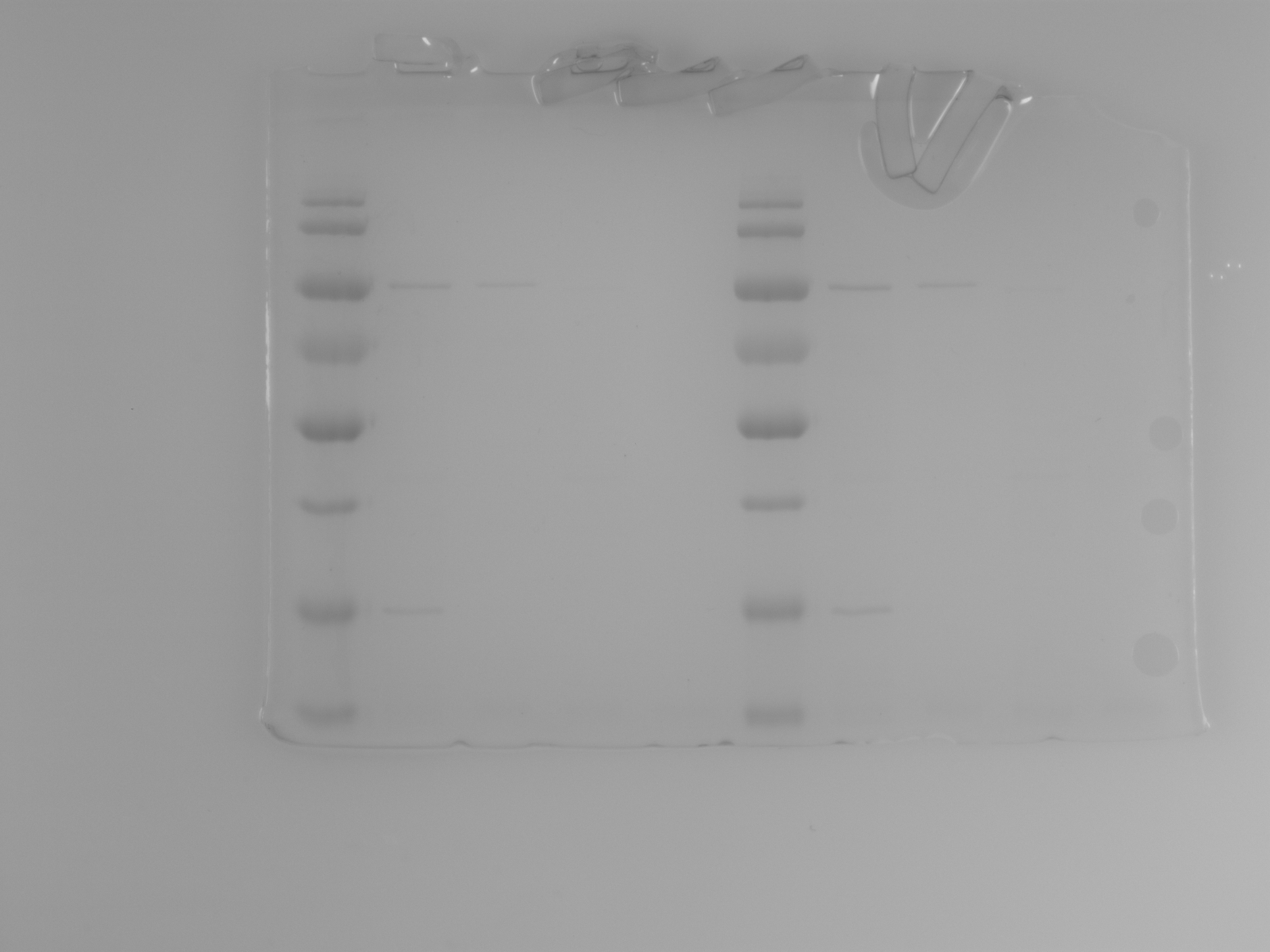

Supplement: Supplementary file 3 — Unprocessed gel for Fig. 2b–f. [file 41589_2023_1539_MOESM3_ESM.zip › Makrydaki_E_SourceDataFig2/Makrydaki_E_SourceDataFig2d.tif]

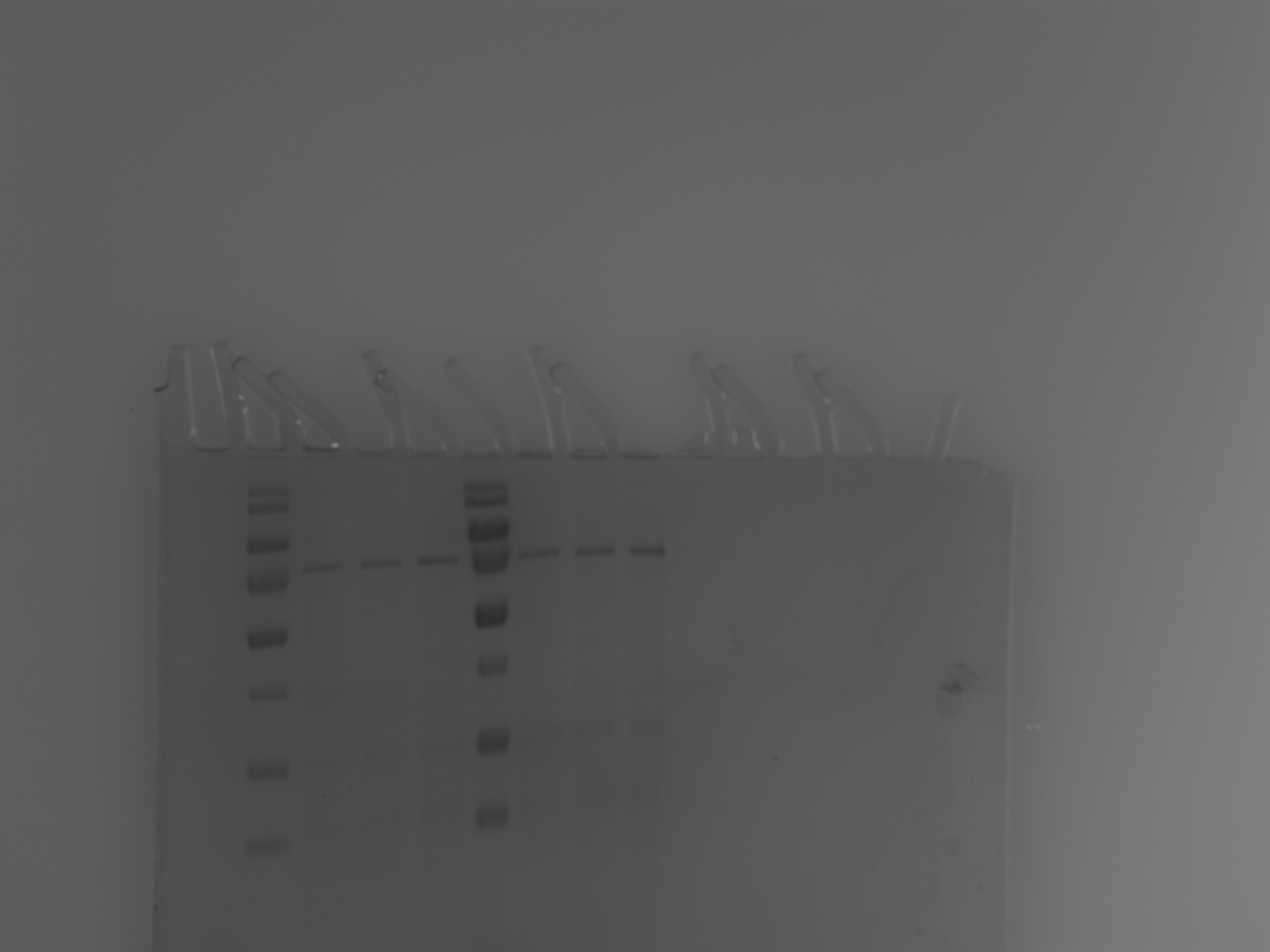

Supplement: Supplementary file 3 — Unprocessed gel for Fig. 2b–f. [file 41589_2023_1539_MOESM3_ESM.zip › Makrydaki_E_SourceDataFig2/Makrydaki_E_SourceDataFig2e.tif]

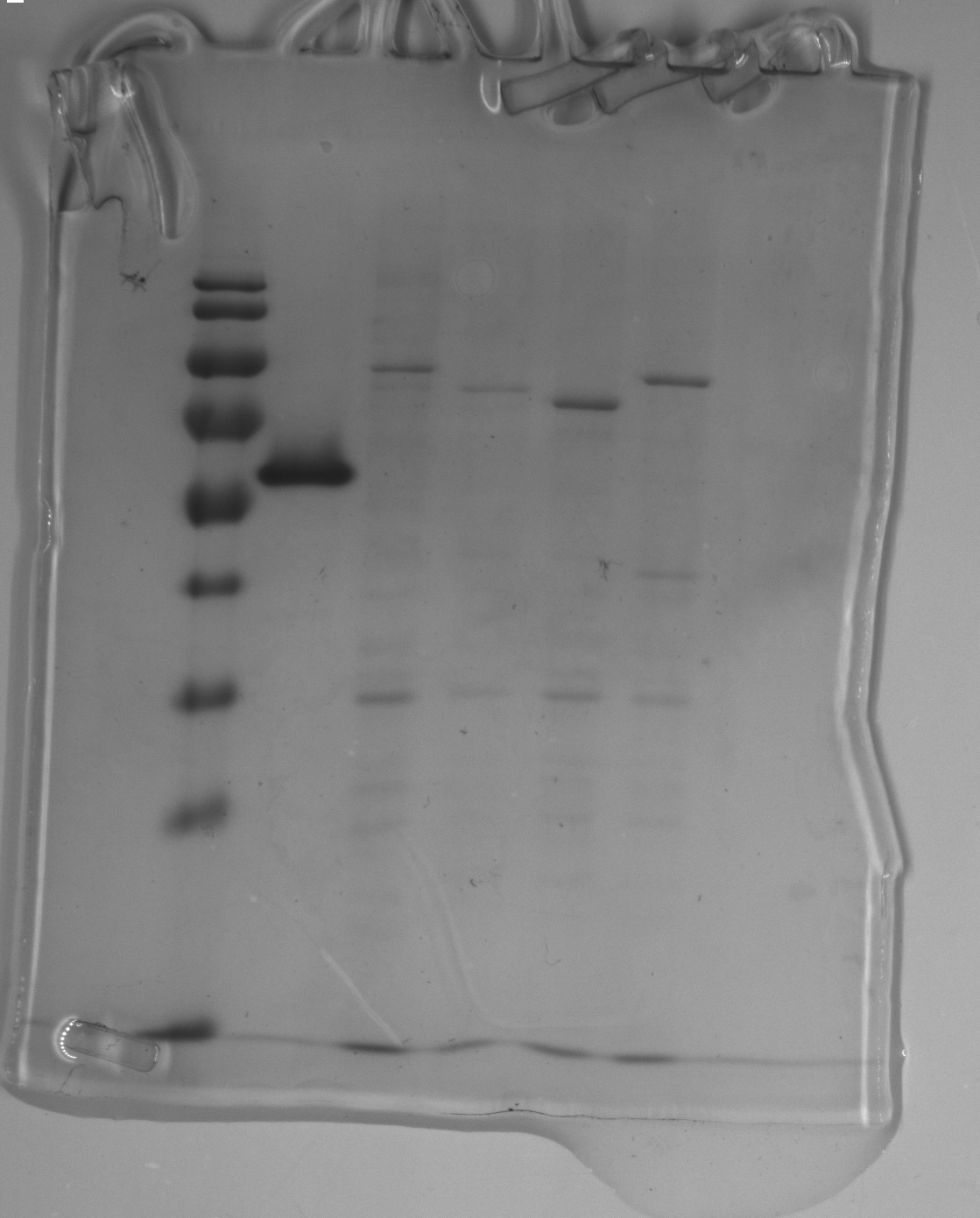

Supplement: Supplementary file 3 — Unprocessed gel for Fig. 2b–f. [file 41589_2023_1539_MOESM3_ESM.zip › Makrydaki_E_SourceDataFig2/Makrydaki_E_SourceDataFig2f.tif]

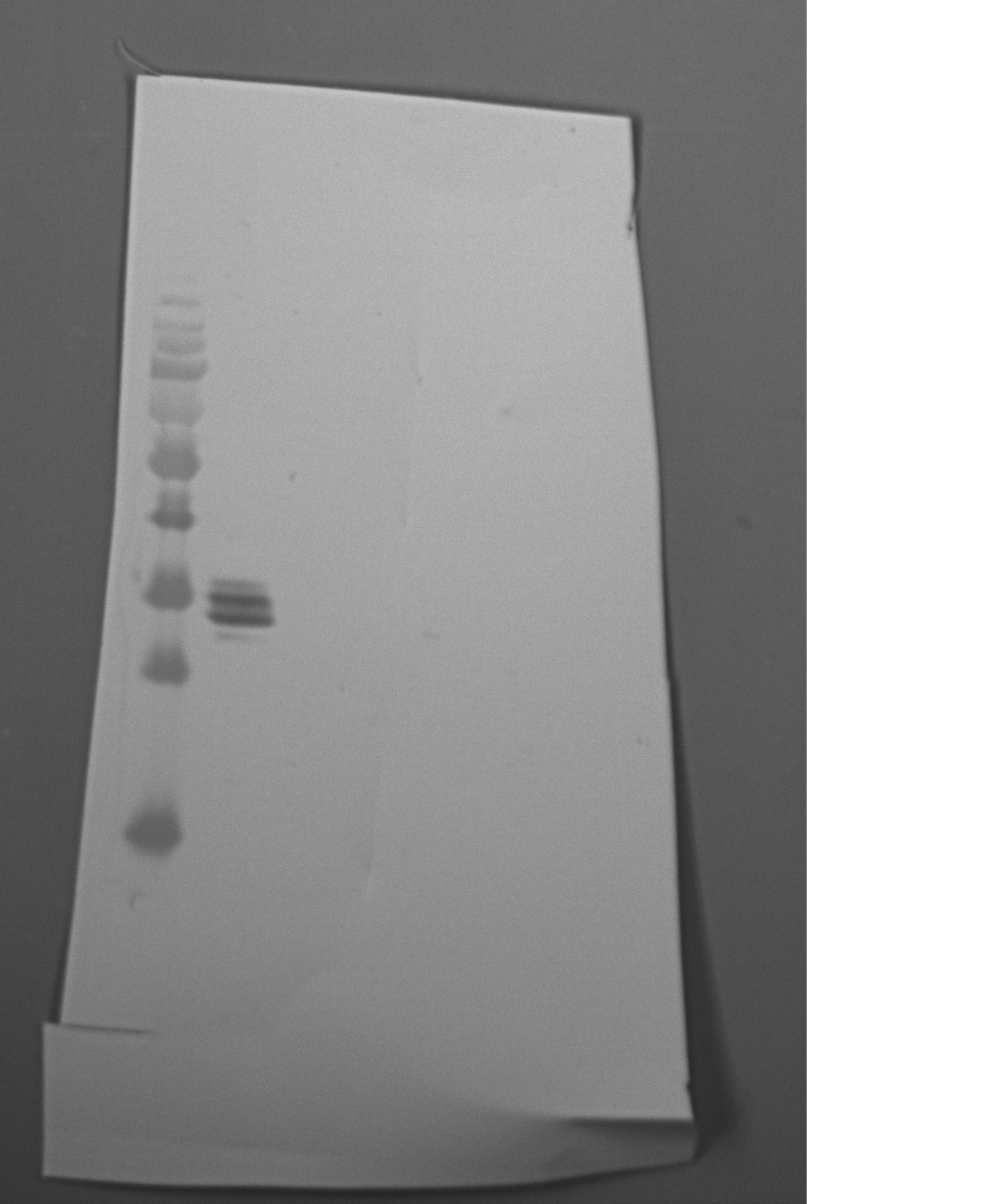

Supplement: Supplementary file 4 — Unprocessed gel for Fig. 3c. [file 41589_2023_1539_MOESM4_ESM.tif]

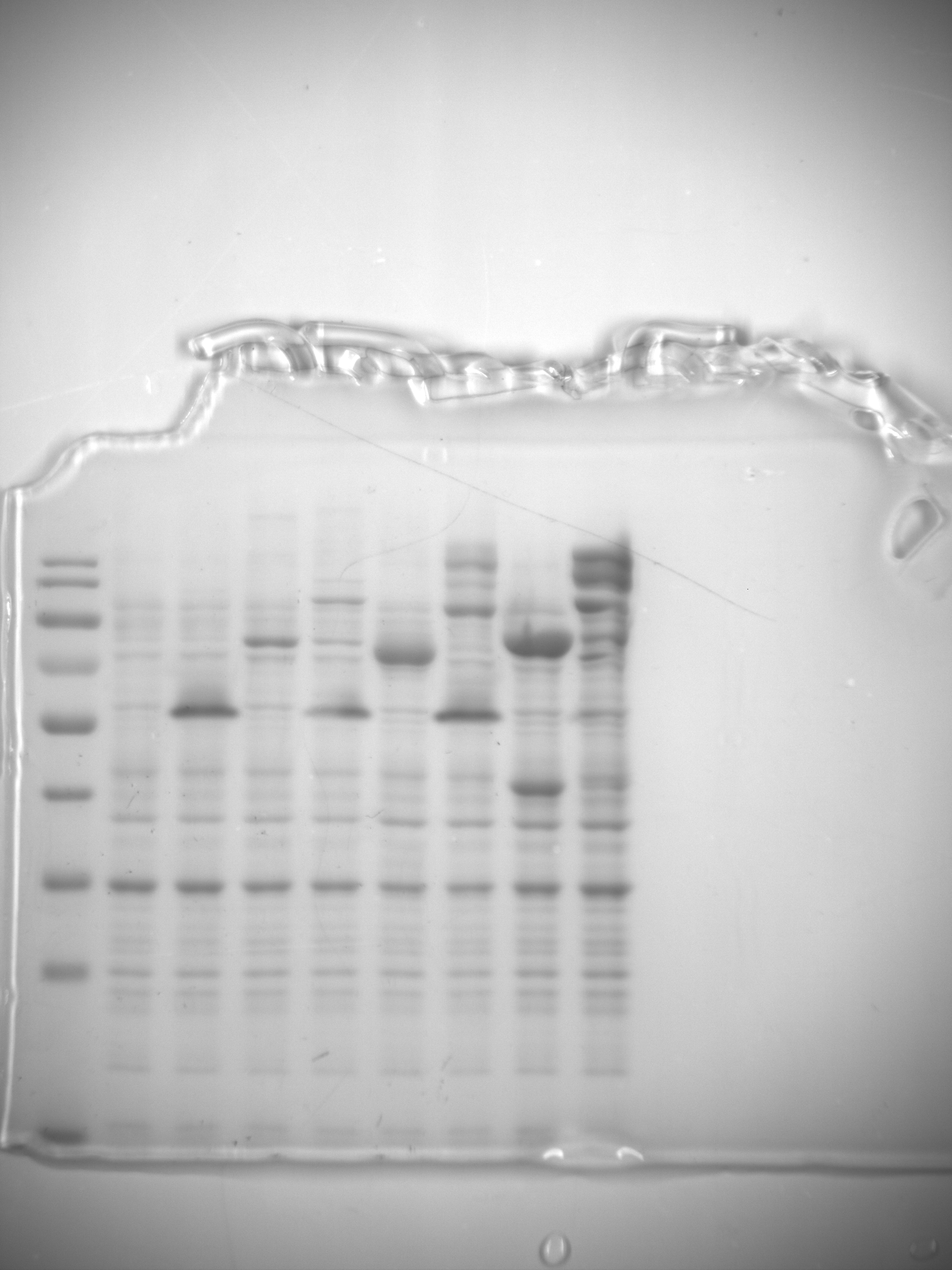

Supplement: Supplementary file 8 — Unprocessed gel. [file 41589_2023_1539_MOESM8_ESM.tif]

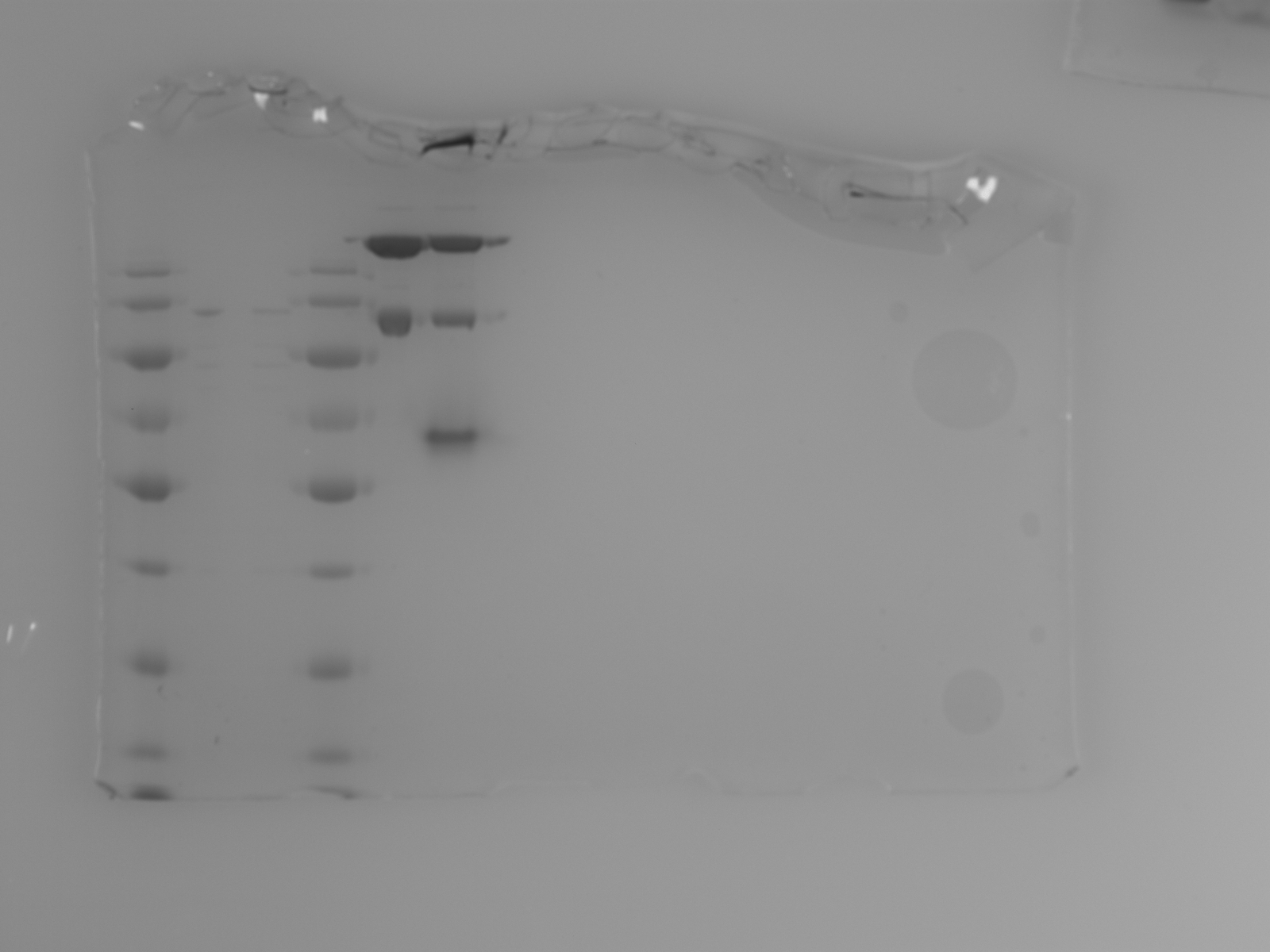

Supplement: Supplementary file 9 — Unprocessed gel. [file 41589_2023_1539_MOESM9_ESM.tif]
