# Supplementary material for: Immobilized enzyme cascade for targeted glycosylation
Source: Nat Chem Biol. 2024 Feb 6;20(6):732–41. doi: 10.1038/s41589-023-01539-4 (PMC11142912; doi:10.1038/s41589-023-01539-4)

CHO h-IgG

Replicate 1

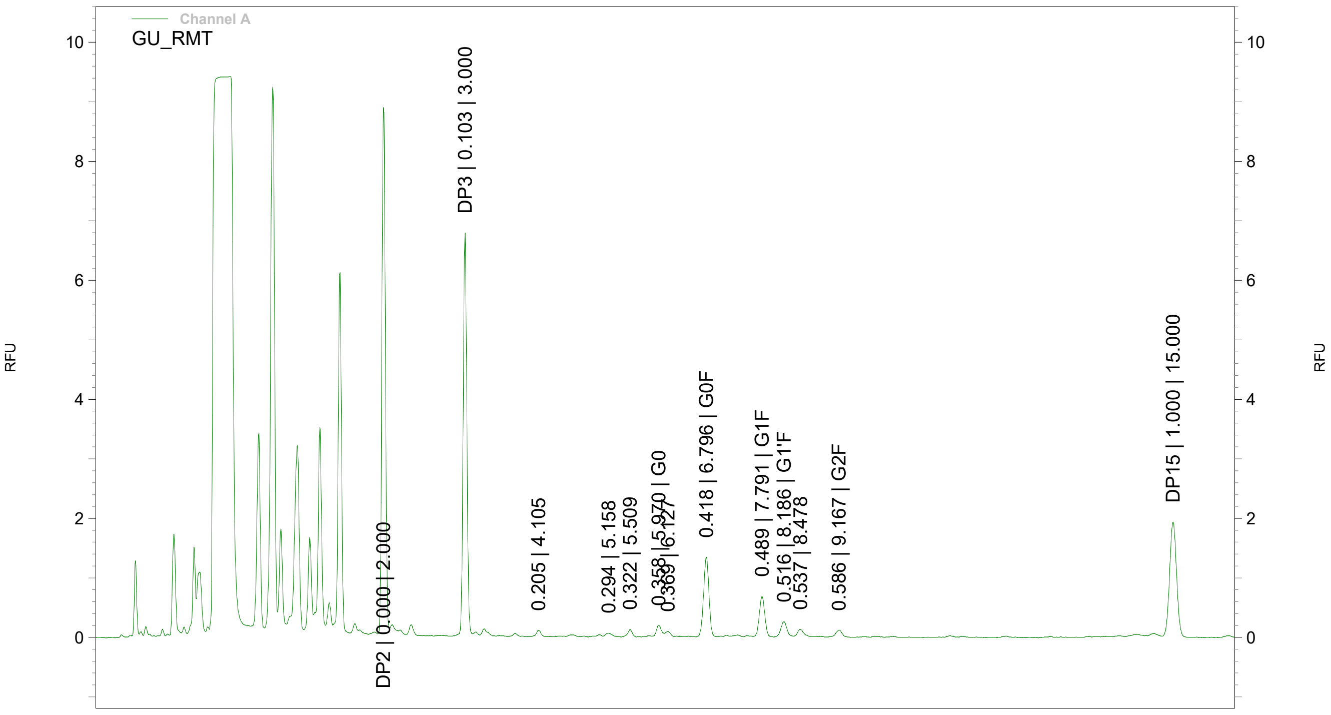

Replicate 2

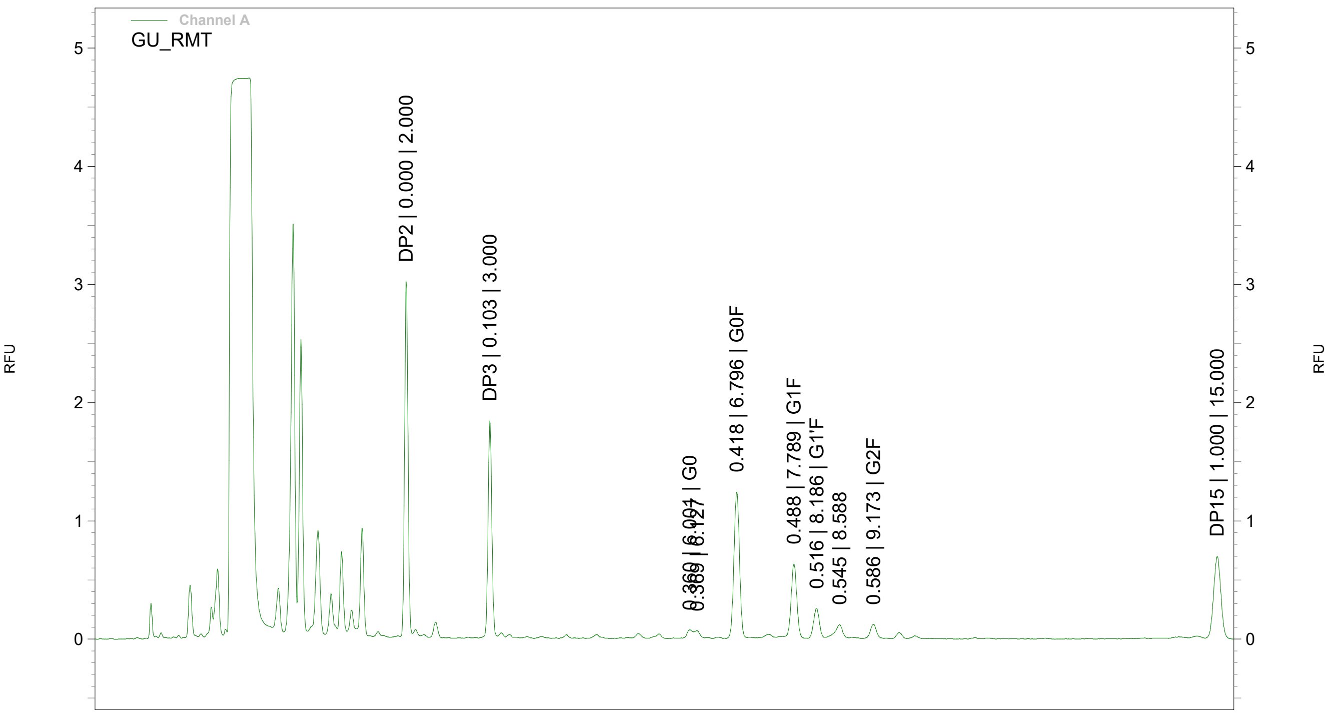

CHO h-IgG + GalT 1st cycle

Replicate 1

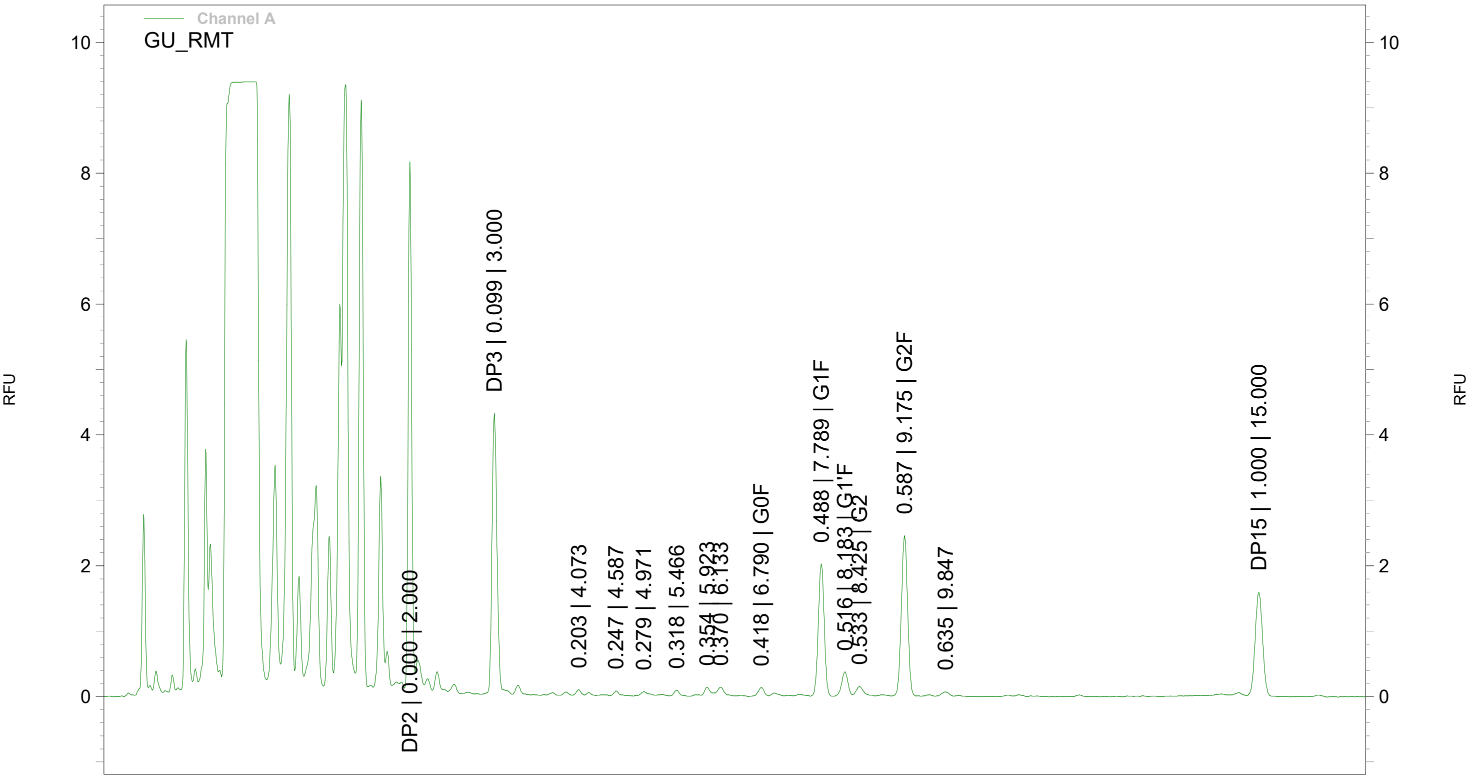

Replicate 2

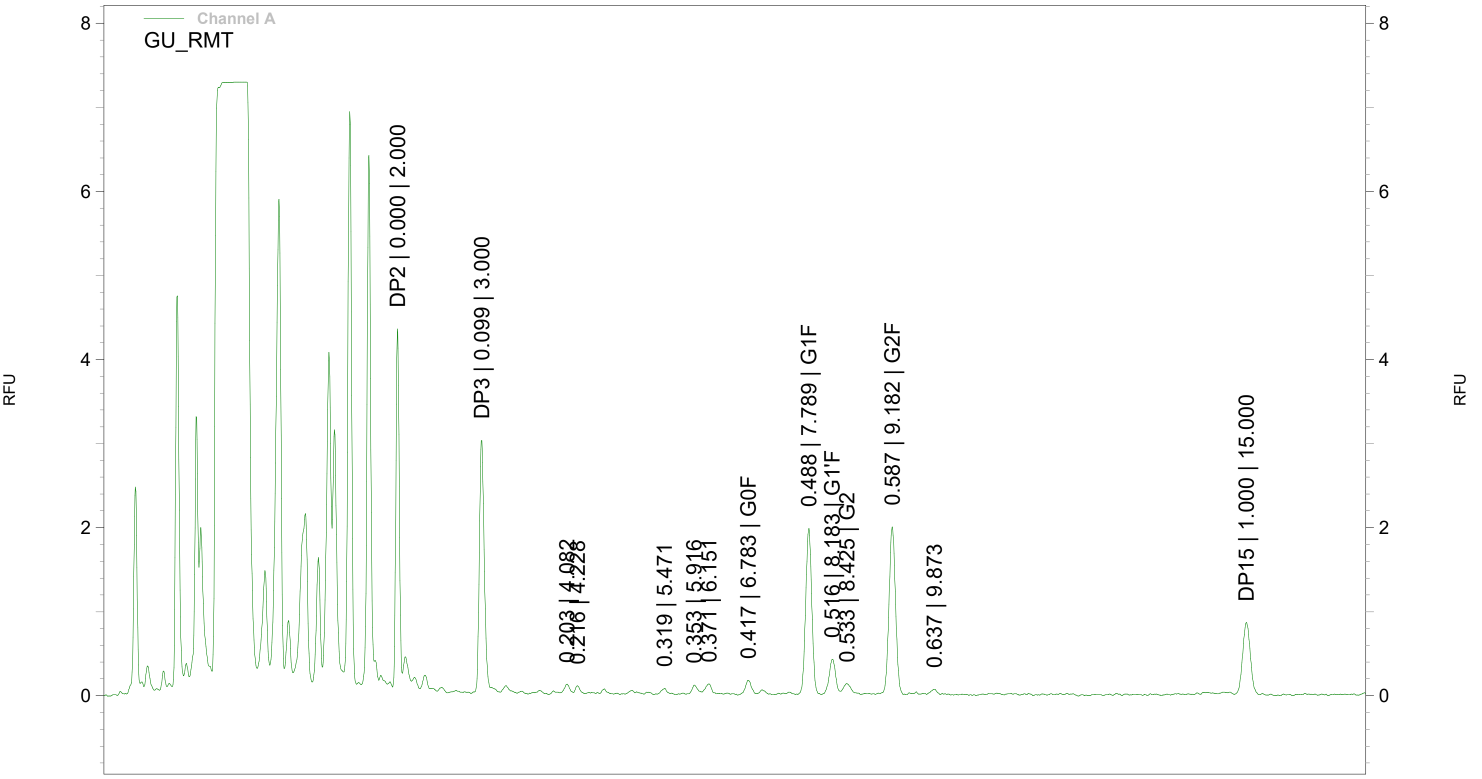

CHO h-IgG + GalT 2nd cycle

Replicate 1

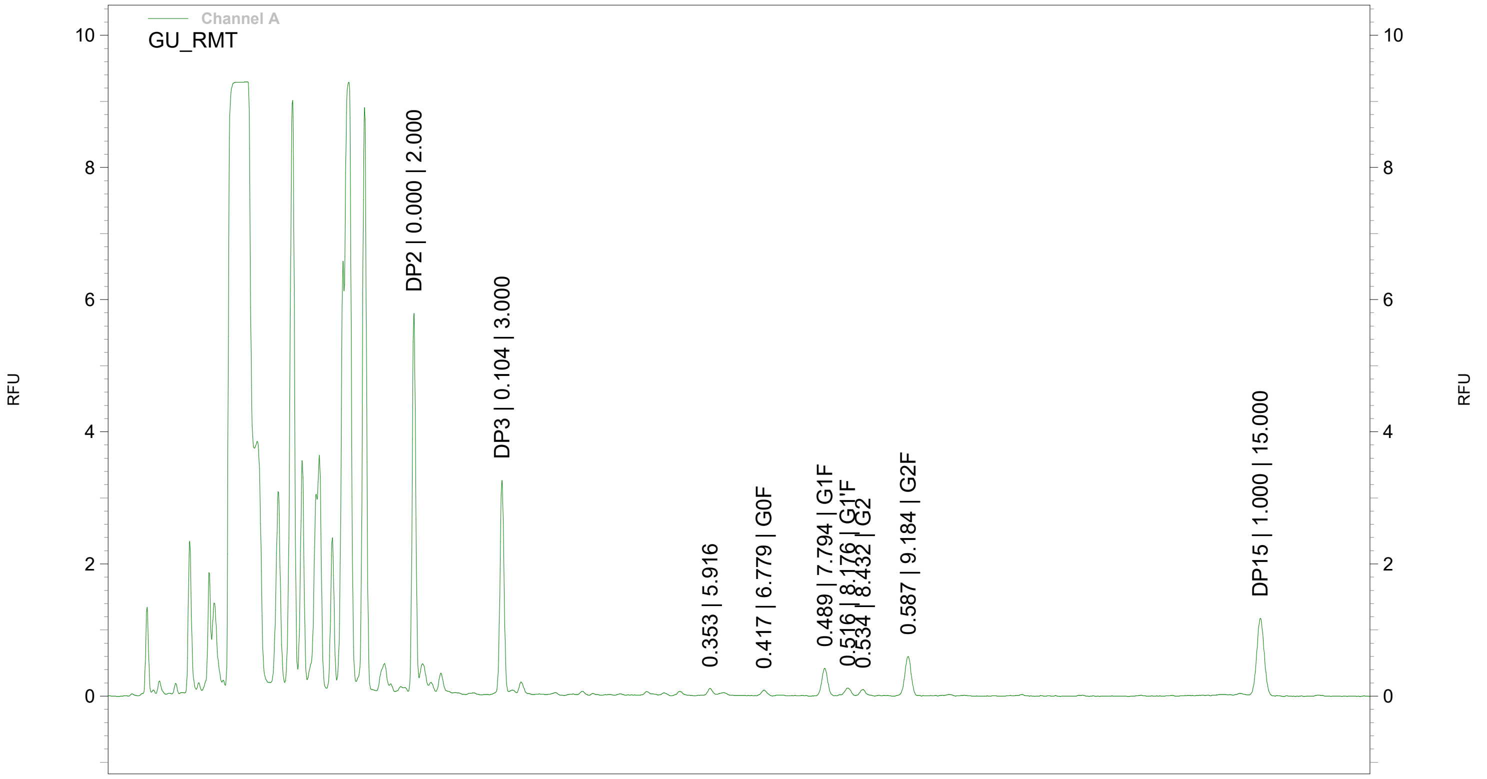

Replicate 2

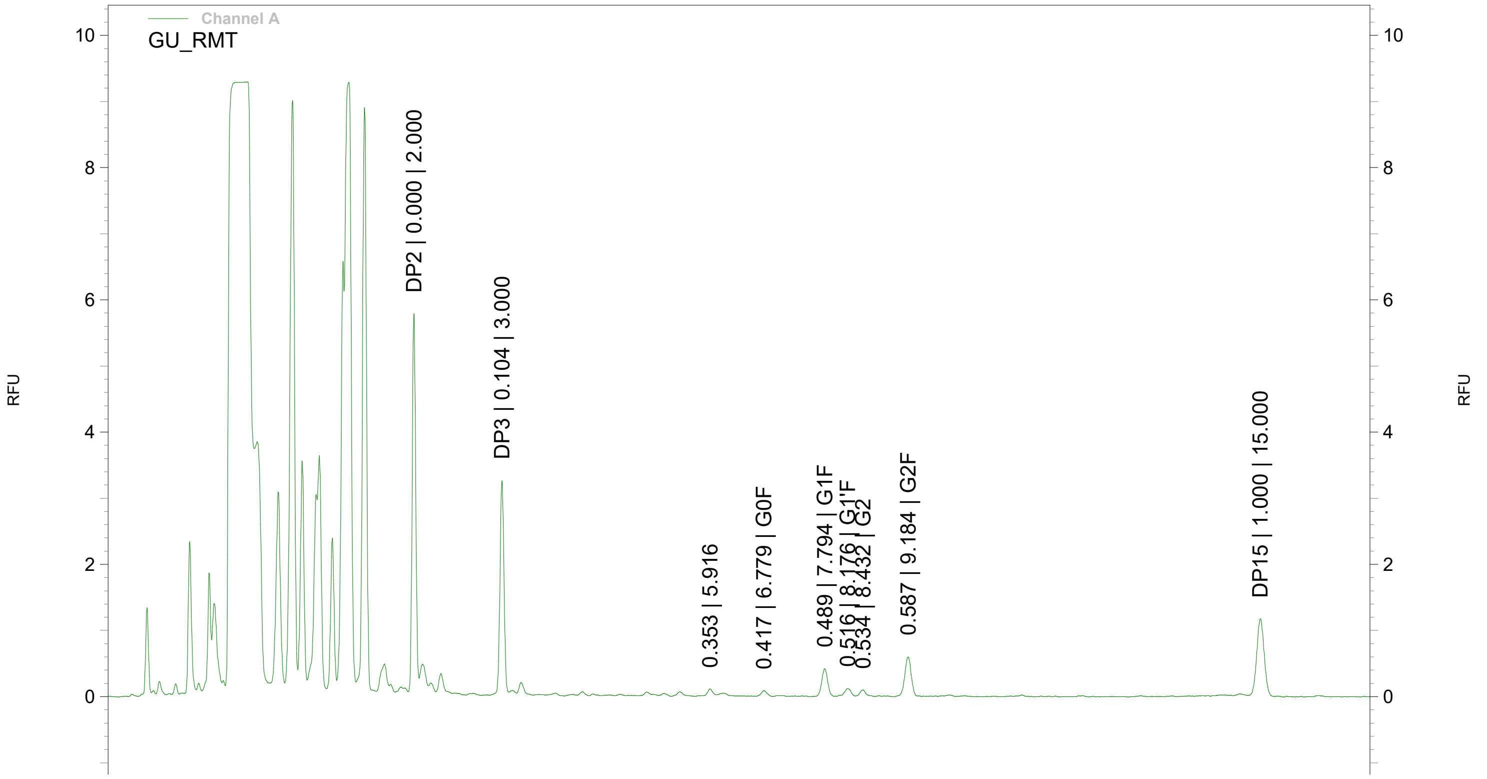

CHO h-IgG + GalT 3rd cycle

Replicate 1

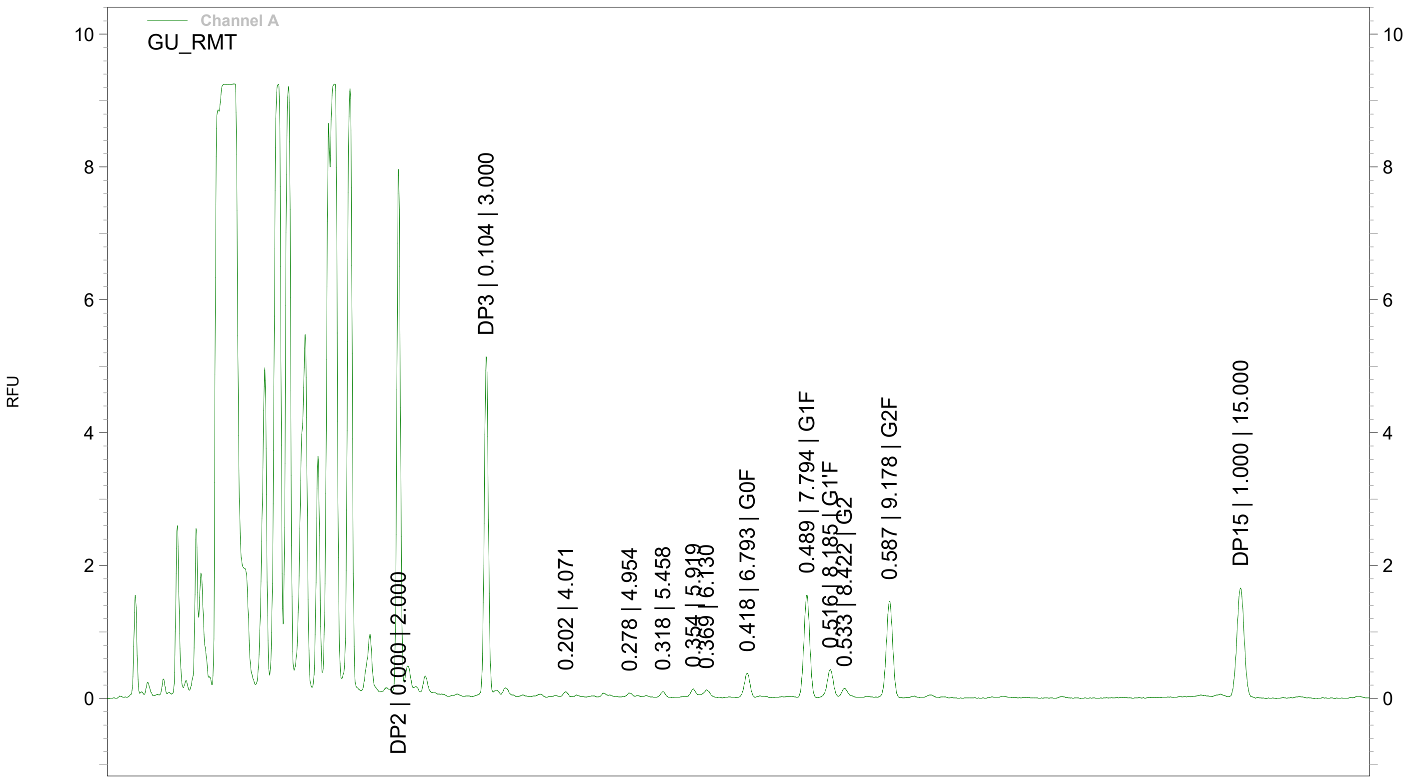

Replicate 2

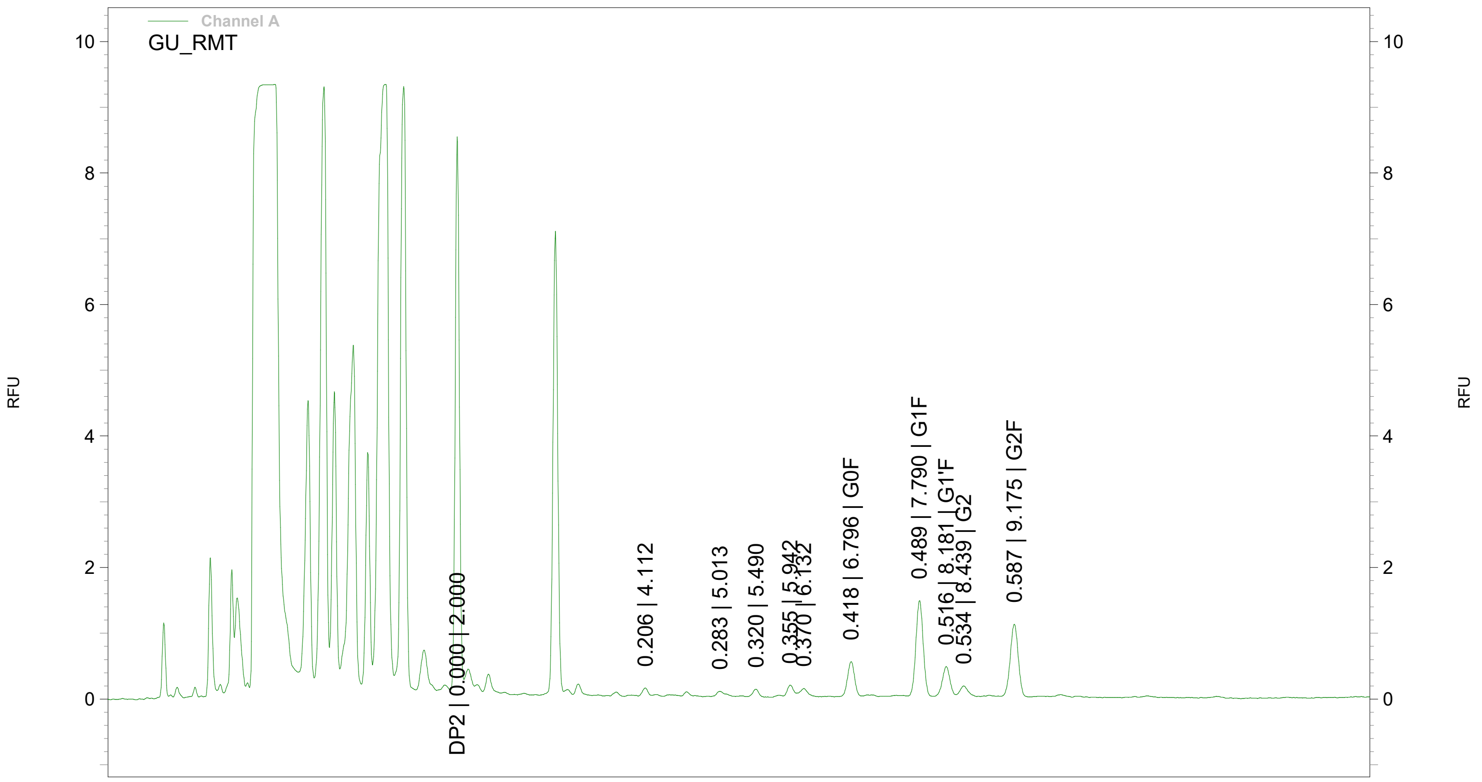

CHO h-IgG + GalT 4th cycle

Replicate 1

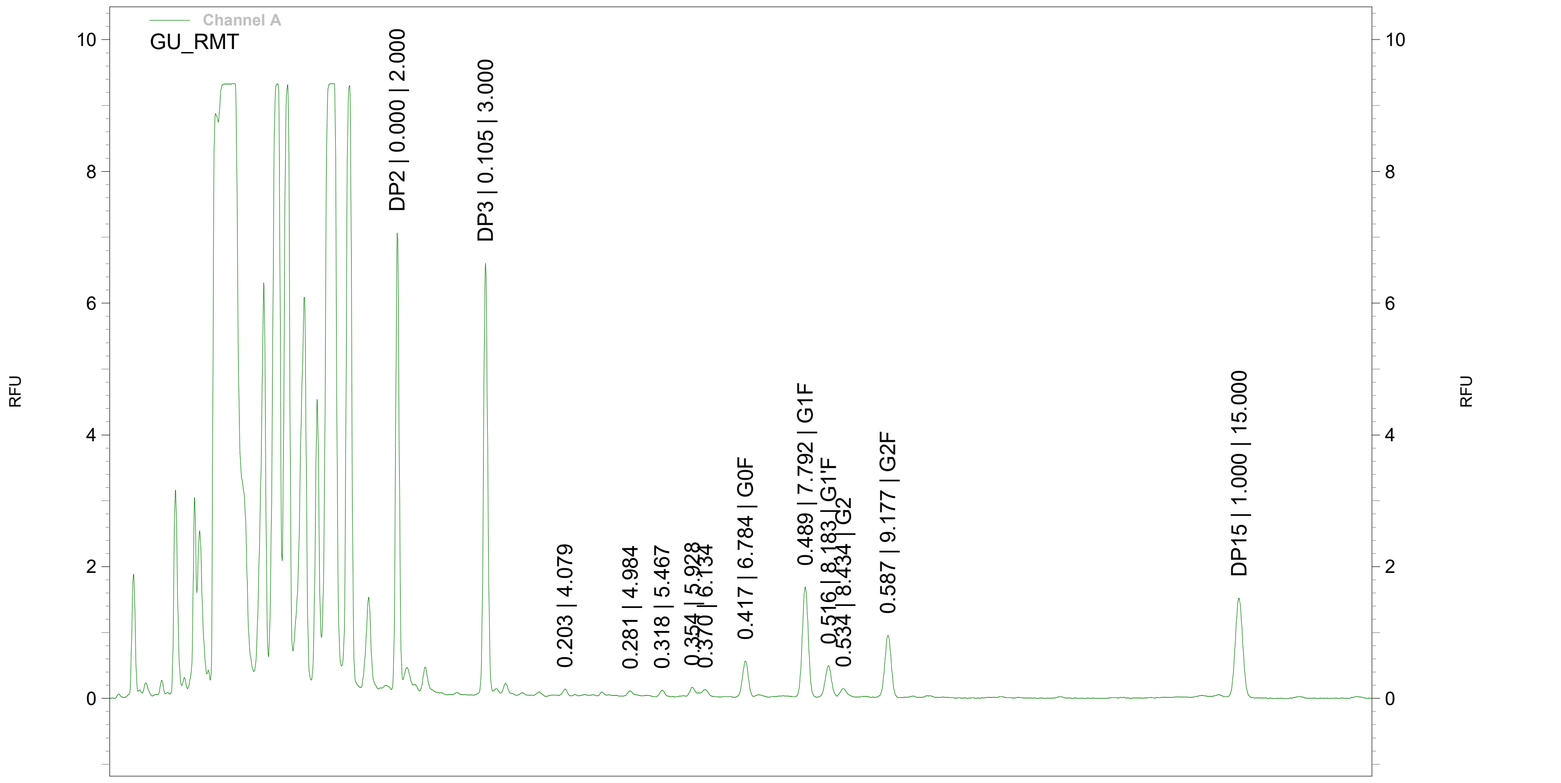

Replicate 2

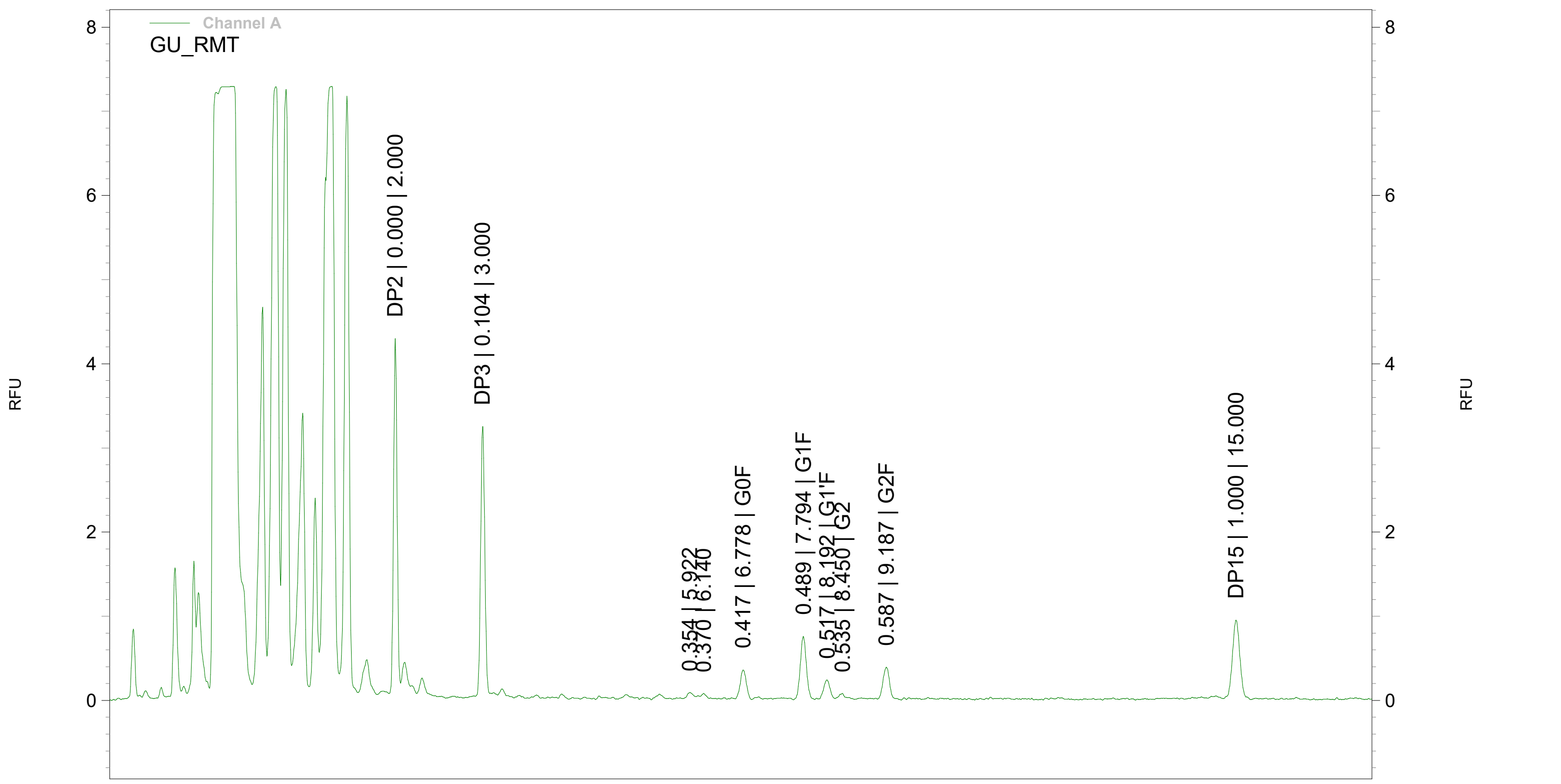

Supplement: Supplementary file 7 — Electropherograms of CE data. [file 41589_2023_1539_MOESM7_ESM.pdf]
